# COVID-19 Vaccination Willingness and Reasons for Vaccine Refusal

**DOI:** 10.1001/jamanetworkopen.2023.37909

**Published:** 2023-10-19

**Authors:** Phyllis Lun, Ke Ning, Yishan Wang, Tiffany S. W. Ma, Francis P. Flores, Xiao Xiao, Mythily Subramaniam, Edimansyah Abdin, Linwei Tian, Tim K. Tsang, Kathy Leung, Joseph T. Wu, Benjamin J. Cowling, Gabriel M. Leung, Michael Y. Ni

**Affiliations:** 1 School of Public Health, LKS Faculty of Medicine, The University of Hong Kong, Hong Kong Special Administrative Region, China; 2 Research Division, Institute of Mental Health, Singapore; 3 Saw Swee Hock School of Public Health, National University of Singapore, Singapore; 4 World Health Organization Collaborating Centre for Infectious Disease Epidemiology and Control, School of Public Health, The University of Hong Kong, Hong Kong Special Administrative Region, China; 5 Laboratory of Data Discovery for Health, Hong Kong Science Park, Hong Kong Special Administrative Region, China; 6 State Key Laboratory of Brain and Cognitive Sciences, The University of Hong Kong, Hong Kong Special Administrative Region, China; 7 Urban System Institute, The University of Hong Kong, Hong Kong Special Administrative Region, China

## Abstract

**Importance:**

Hong Kong was held as an exemplar for pandemic response until it recorded the world’s highest daily COVID-19 mortality, which was likely due to vaccine refusal. To prevent this high mortality in future pandemics, information on underlying reasons for vaccine refusal is necessary.

**Objectives:**

To track the evolution of COVID-19 vaccination willingness and uptake from before vaccine rollout to mass vaccination, to examine factors associated with COVID-19 vaccine refusal and compare with data from Singapore, and to assess the population attributable fraction for vaccine refusal.

**Design, Setting, and Participants:**

This cohort study used data from randomly sampled participants from 14 waves of population-based studies in Hong Kong (February 2020 to May 2022) and 2 waves of population-based studies in Singapore (May 2020 to June 2021 and October 2021 to January 2022), and a population-wide registry of COVID-19 vaccination appointments. Data were analyzed from February 23, 2021, to May 30, 2022.

**Exposures:**

Trust in COVID-19 vaccine information sources (ie, health authorities, physicians, traditional media, and social media); COVID-19 vaccine confidence on effectiveness, safety, and importance; COVID-19 vaccine misconceptions on safety and high-risk groups; political views; and COVID-19 policies (ie, workplace vaccine mandates and vaccine pass).

**Main Outcomes and Measures:**

Primary outcomes were the weighted prevalence of COVID-19 vaccination willingness over the pandemic, adjusted incidence rate ratios, and population attributable fractions of COVID-19 vaccine refusal. A secondary outcome was change in daily COVID-19 vaccination appointments.

**Results:**

The study included 28 007 interviews from 20 waves of longitudinal data, with 1114 participants in the most recent wave (median [range] age, 54.2 years [20-92] years; 571 [51.3%] female). Four factors—mistrust in health authorities, low vaccine confidence, vaccine misconceptions, and political views—could jointly account for 82.2% (95% CI, 62.3%-100.0%) of vaccine refusal in adults aged 18 to 59 years and 69.3% (95% CI, 47.2%-91.4%) of vaccine refusal in adults aged 60 years and older. Workplace vaccine mandates were associated with 62.2% (95% CI, 9.9%-139.2%) increases in daily COVID-19 vaccination appointments, and the Hong Kong vaccine pass was associated with 124.8% (95% CI, 65.9%-204.6%) increases in daily COVID-19 vaccination appointments.

**Conclusions and Relevance:**

These findings suggest that trust in health authorities was fundamental to overcoming vaccine hesitancy. As such, engendering trust in health care professionals, experts, and public health agencies should be incorporated into pandemic preparedness and response.

## Key Points

**Question:**

What were the reasons for COVID-19 vaccine refusal in Hong Kong, and what policy measures may be associated with higher vaccine uptake?

**Findings:**

This cohort study used data from 28 007 interviews over 20 waves, including 1114 participants in the latest wave in 2022, found that 75.0% of vaccine refusal could be attributable to mistrust in health authorities, low vaccine confidence, misconceptions, and political views. The vaccine pass policy was associated with an increase in vaccination appointments.

**Meaning:**

These findings suggest that building trust in health authorities, promoting vaccine confidence, and countering misinformation may be fundamental for better preparedness and response to future pandemics.

## Teaser

This cohort study uses interview data from Hong Kong residents to assess COVID-19 vaccination willingness and uptake and assess factors associated with vaccine refusal.

## Introduction

Hong Kong was held as an exemplar of pandemic response until the city recorded the world’s highest daily COVID-19 mortality to date, reaching 39.3 deaths per million people.^1,2^ In comparison, the highest levels of daily COVID-19 mortality were 22.1 deaths per million people in the United Kingdom and 13.0 deaths per million people in the US.^2^ While Hong Kong’s cumulative COVID-19 deaths per capita remain lower than the UK and US, it has far exceeded high-income economies in Asia-Pacific (eFigure 1 in Supplement 1).^2^ A probable key reason for Hong Kong’s COVID-19 death toll was low vaccination coverage among older adults.^3^ Specifically, when Hong Kong was hit with the Omicron BA.2 variant, 82.4% of adults aged 80 years and older were unvaccinated or had received only 1 dose. Equivalent figures in other jurisdictions were 2.9% in New Zealand, 9.0% in Singapore, and 6.7% in England (eTable 1 in Supplement 1). Understanding COVID-19 vaccine refusal could help prevent Hong Kong’s high mortality from repeating elsewhere.^4^ However, the causes of vaccine refusal in Hong Kong are poorly understood.^3^

One potential explanation for Hong Kong’s vaccine refusal is that the city attained sustained periods of so-called *zero-COVID* (ie, no local COVID-19 cases), which may have resulted in vaccine complacency.^4,5^ However, other jurisdictions that had implemented elimination strategies (eg, Singapore, Australia, and New Zealand) have attained excellent vaccination coverage in their populations (eFigure 2 in Supplement 1). Another potential explanation is that Hong Kong experienced major social upheaval immediately before COVID-19. The 2019 to 2020 social unrest in relation to a proposed extradition bill was the largest social unrest in Hong Kong in more than half a century.^6^ Hong Kong was deeply divided and mistrustful of official authorities from the outset of the pandemic.^7^ Therefore, this unique phenomenon of successive population shocks in Hong Kong could be leveraged as a natural experiment to study the role of mistrust and political views in vaccine refusal.^4,6,7^

In this study, we used 20 waves of data from a prospective cohort study and a population-wide registry of all COVID-19 vaccination appointments to address 3 objectives. First, we tracked the evolution of vaccination willingness and uptake from before vaccine rollout to mass vaccination. Second, we examined the determinants of COVID-19 vaccine refusal in Hong Kong. We then compared key factors associated with vaccine refusal between Hong Kong and Singapore, given that Singapore launched one of the most successful vaccination programs worldwide.^2^ Finally, we assessed the population attributable fractions of vaccine refusal associated with key factors.

## Methods

This cohort study received ethical approval from the Institutional Review Board of The University of Hong Kong, Hospital Authority Hong Kong West Cluster, and the Domain Specific Review Board, National Healthcare Group, Singapore. Verbal informed consent was obtained from all study participants in Hong Kong and Singapore. This study followed the Strengthening the Reporting of Observational Studies in Epidemiology (STROBE) reporting guideline for cohort studies.

### Study Design and Participants

The sample was drawn from the FAMILY Cohort, a prospective population-based cohort study in Hong Kong.^8^ A total of 18 045 adults and adolescents (aged ≥15 years) and 1488 children (aged 10-14 years) were enrolled using stratified random sampling between 2009 and 2011 (wave 1) and followed up between 2011 and 2014 (wave 2). We subsequently randomly sampled participants from wave 2 and followed up with them over the last decade (≥1000 participants for each wave), including during the 2014 Occupy Central protests (waves 3-4) and 2019 to 2020 social unrest (waves 5-6). During the COVID-19 pandemic (waves 7-20), we interviewed the panel via telephone 14 times from February 2020 to May 2022. This encompassed the time period before the advent of COVID-19 vaccines (waves 7-13), during the vaccine rollout (waves 14-17), and during and after the Omicron wave that led to the major surge in COVID-19 mortality (waves 18-20). The survey periods and sample sizes for each wave during the 14-year prospective cohort study are shown in eFigure 3 in Supplement 1. Response and cooperation rates were calculated according to prevailing accepted standards (eMethods in Supplement 1).^9^

We compared key factors associated with vaccine hesitancy in Hong Kong and Singapore. The population-based sample in Singapore (baseline: 1129 participants; follow-up: 500 participants) was drawn from the second Singapore Mental Health Study in 2016, a nationwide epidemiological study that recruited participants randomly from an administrative database of all Singapore residents (eMethods in Supplement 1).^10^ We obtained the daily number of COVID-19 vaccination appointments among Hong Kong adults from February 2021 to May 2022 from the official population-wide registry that includes a total of 5 050 999 vaccination appointments.

### Study Outcomes

The primary outcomes were weighted prevalence of COVID-19 vaccination (waves 11-20) and adjusted incidence rate ratios and population attributable fractions of COVID-19 vaccine refusal. Willingness to vaccinate against COVID-19 (waves 11-20) assessed as received at least 1 dose of vaccine, made a vaccination appointment, or expressed an intention to vaccinate. COVID-19 vaccine refusal was defined as having no intention to receive COVID-19 vaccines.^11,12^ The secondary outcome was change in daily COVID-19 vaccination appointments.

### Exposures

Political views were assessed during the 2019 to 2020 social unrest by whether participants were for, against, or neutral toward the extradition bill. These were classified as proestablishment, nonestablishment, and neutral, respectively.^6^ Political participation was assessed during the 2014 Occupy Central protests (waves 3-4) by whether individuals participated or visited the protest sites. This served as a proxy for political views.^13^

Trust in COVID-19 vaccine information sources in Hong Kong (waves 15-20) and in Singapore was assessed by the level of trust in the World Health Organization (WHO), government health authorities, physicians, academics, and traditional and social media platforms as information sources for COVID-19 vaccines. COVID-19 vaccine confidence (waves 11-18 and 20) was assessed using 3 statements on the perceived effectiveness, safety, and importance of COVID-19 vaccines that were based on the Vaccine Confidence Index.^14,15^ COVID-19 vaccine misconceptions about older adults, chronic diseases, and vaccine safety were assessed in Hong Kong (waves 15-20) and Singapore (eMethods in Supplement 1). Details of outcomes and exposures can be found in eTable 2 in Supplement 1.

### Statistical Analysis

#### Vaccination Willingness From Before Vaccine Rollout to Mass Vaccination

We estimated the weighted prevalence of vaccination willingness over the COVID-19 pandemic. To account for demographic differences between each wave of the survey sample and the Hong Kong adult population, we applied both poststratification weighting and raking to the data in all analyses (eMethods in Supplement 1). Cohen *w* was calculated to assess the sociodemographic differences between our sample and the Hong Kong adult population (eMethods in Supplement 1).^16^ We used interrupted time series analysis to evaluate the associations between policy measures and vaccine uptake (eMethods in Supplement 1).

#### Determinants of COVID-19 Vaccine Refusal

We used robust Poisson regression to examine the associations of political views with trust in COVID-19 vaccine information sources, COVID-19 vaccine confidence, vaccine misconceptions, and COVID-19 vaccine refusal. In all models, we adjusted for sociodemographic characteristics (ie, age, sex, educational attainment, marital status, employment status, and household income). We additionally adjusted for political views during 2019 to 2020 social unrest when examining the association of trust in vaccine information sources, vaccine confidence, and vaccine misconceptions with vaccine refusal. As it may not be appropriate to change political beliefs, we therefore conducted secondary analyses to identify potentially modifiable mediators. Specifically, we used causal mediation analysis to examine the indirect association of political views with vaccine refusal via trust in information sources, vaccine confidence, and vaccine misconceptions (eMethods in Supplement 1).^17^

#### Potential Gains in Vaccination Willingness

To estimate the joint contribution of determinants to vaccine refusal, we estimated sequential and average population attributable fractions (PAFs) by modeling the determinants simultaneously (eMethods in Supplement 1).^18^ To estimate the individual contribution of determinants to vaccine refusal, we used robust Poisson regression, since it can better handle common outcomes and model misspecification.^19^ We stratified by age (18-59 years and ≥60 years), since low vaccination coverage among older adults was the driving factor associated with high COVID-19 mortality in Hong Kong.^3,20^

We applied multiple imputation to handle incomplete data and pooled the results from 20 imputed data sets using Rubin rules.^21^ *P* values were 2-sided, and statistical significance was set at *P* = .05. All analyses were conducted using R statistical software version 4.1.3 (R Project for Statistical Computing) and Stata/MP statistical software version 17.0 (StataCorp). Data were analyzed from February 23, 2021, to May 30, 2022.

## Results

In total, we conducted 28 007 interviews over 20 waves of longitudinal data. Across follow-ups, the median (range) response rate was 75.7% (70.5%-78.6%) and the median (range) cooperation rate was 63.4% (60.0%-79.4%) (eFigure 3 in Supplement 1). The most recent follow-up included 1114 participants (median [range] age, 54.2 [20-92] years; 571 [51.3%] female). Sociodemographic differences between the most recent follow-up (weighted wave 20) and the 2016 Hong Kong population per the latest census data were small (Cohen *w* < 0.1) (eTable 3 in Supplement 1).

### Vaccination Willingness From Before Vaccine Rollout to Mass Vaccination

**Figure 1.**
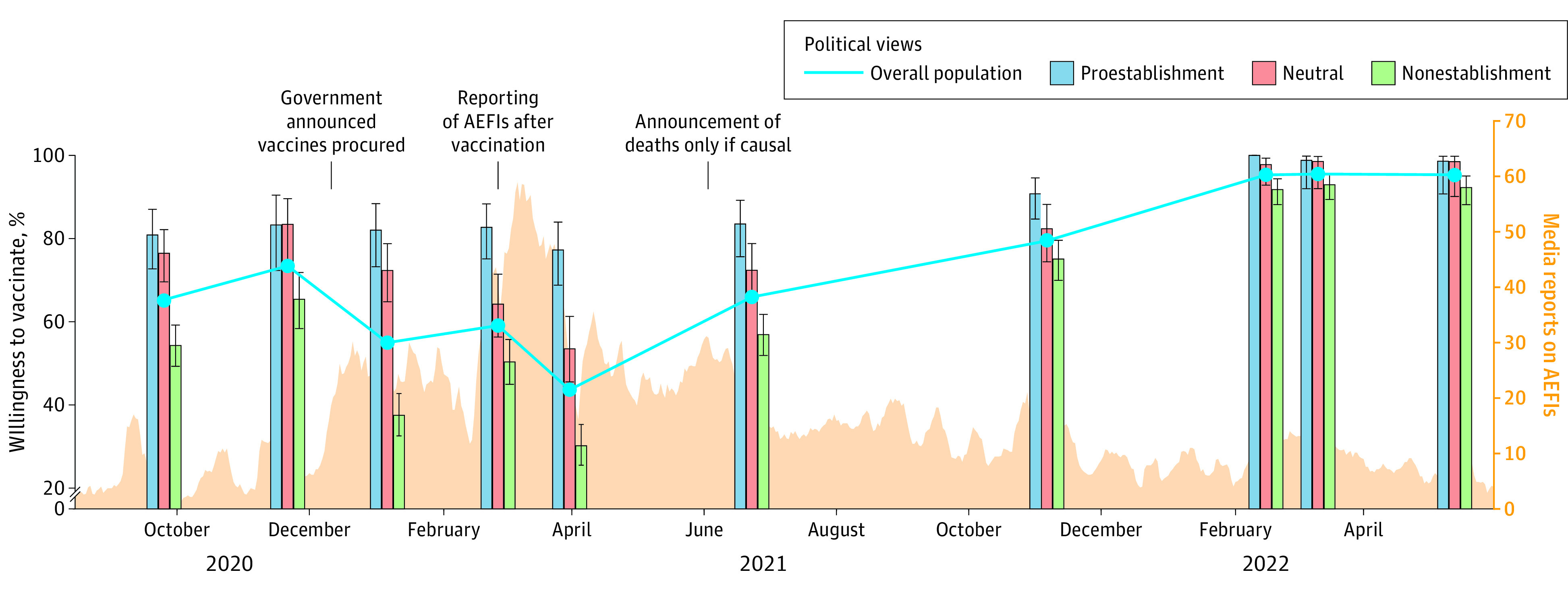
Trends in Vaccination Willingness and Media Reports on Adverse Events Following COVID-19 Immunization (AEFIs)

In 2020, approximately two-thirds (65.3% [95% CI, 61.7%-68.6%]) of adults in Hong Kong said they would be willing to vaccinate when a vaccine became available. Vaccination willingness then increased to 73.4% (95% CI, 69.0%-77.4%) of individuals, which coincided with positive results from COVID-19 vaccine trials and a local COVID-19 outbreak. However, after the Hong Kong government announced that vaccines from Germany, Mainland China, and the UK were procured and that individuals could not choose which vaccine to receive (a decision that was reversed 12 days later), vaccination willingness dropped to 55.0% (95% CI, 51.3%-58.6%) of individuals.^22,23^ The launch of the vaccination program and a peak in media reports on adverse events following COVID-19 immunization (AEFIs) corresponded with a decline in vaccination willingness to its lowest level (43.6% [95% CI, 40.0%-47.3%]) (Figure 1).

**Figure 2.**
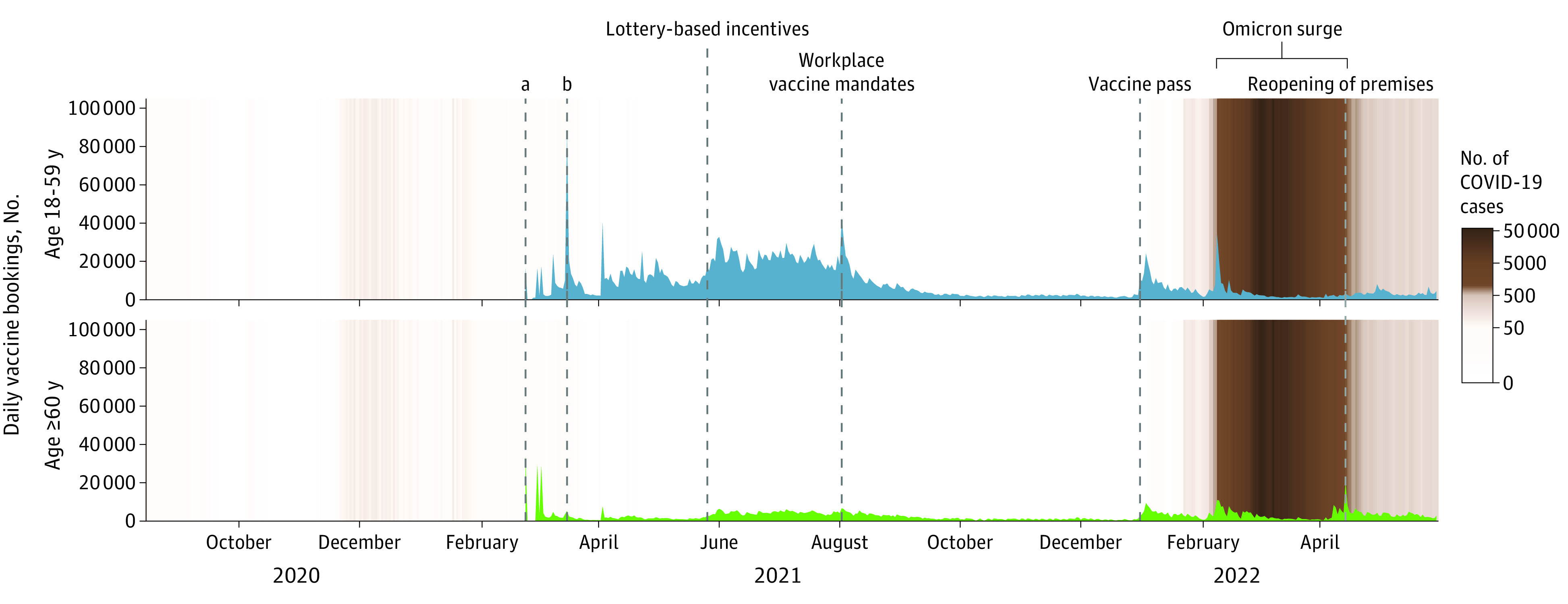
Daily Appointments of COVID-19 Vaccination (Primary Series) for Individuals Aged 18 to 59 Years (Top Panel) and ≥60 Years (Bottom Panel)

During periods with no major public policies or events, mean (SD) daily numbers of vaccination appointments were 5636 (5018) appointments per day among adults aged 18 to 59 years and 1857 (1192) for adults aged 60 years and older (Figure 2). In adults aged 18 to 59 years, workplace vaccine mandates were associated with an increase in daily COVID-19 vaccination appointments by 73.7% (95% CI, 20.2%-150.9%), and the vaccine pass in Hong Kong was associated with an increase of 130.9% (95% CI, 72.2%-209.7%) (eTable 4 in Supplement 1). Vaccination appointments increased by 43.8% (95% CI, 6.1%-94.8%) during the Omicron surge. Lottery-based incentives were not associated with changes in vaccination appointments. In older adults (age ≥60 years), the vaccine pass was associated with a 93.5% (95% CI, 32.5%-182.4%) increase in vaccination appointments, and subsequent reopening of premises under the vaccine pass was associated with an 111.6% (95% CI, 47.5%-203.6%) increase. The Omicron surge was associated with increasing vaccination appointments in older adults by 106.8% (95% CI, 42.2%-200.6%). Lottery-based incentives and workplace mandates were not associated with changes in vaccination appointments among older adults.

### Factors Associated With COVID-19 Vaccine Refusal

#### Political Views

**Figure 3.**
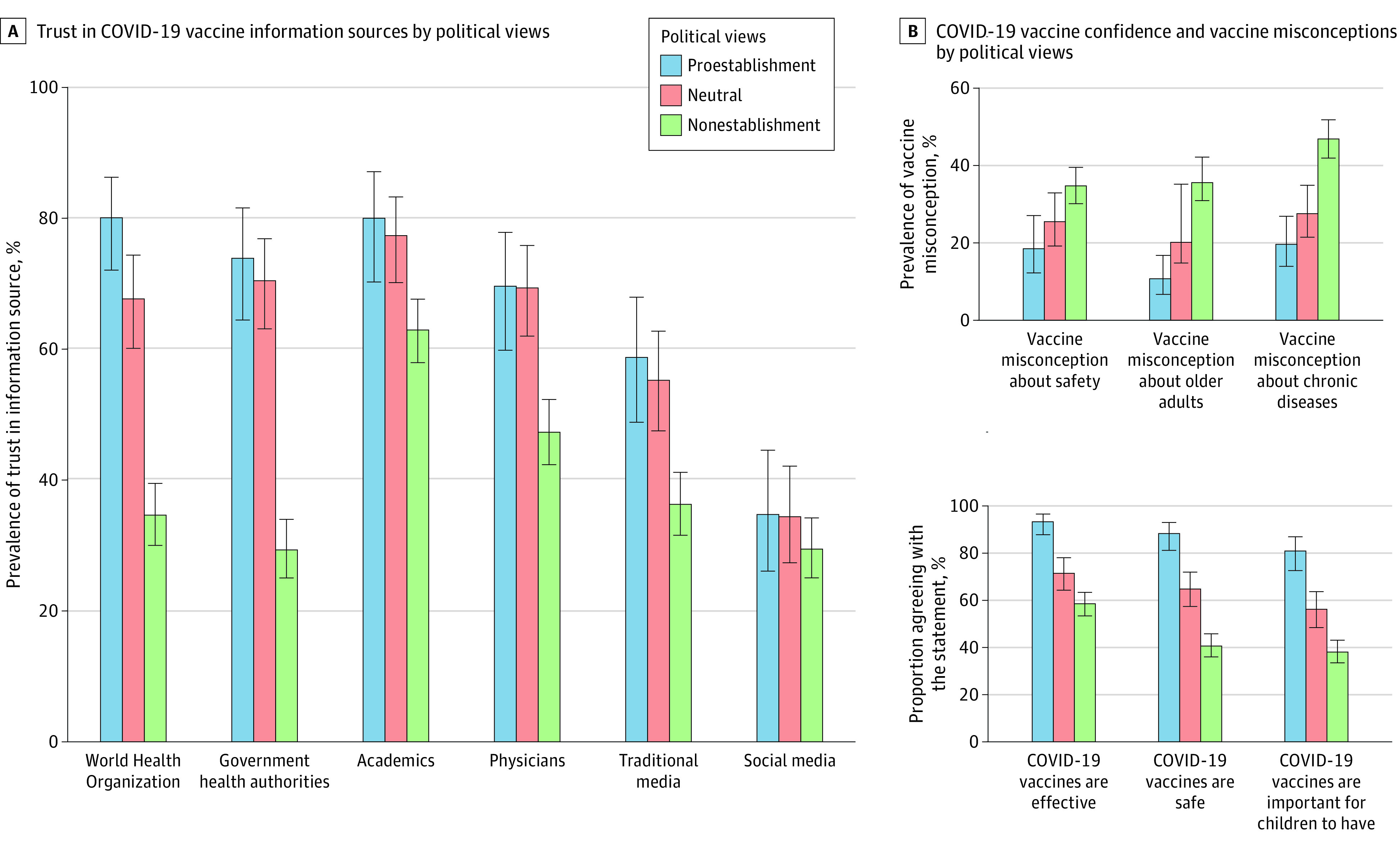
Trust in Information Sources, Vaccine Confidence, and Vaccine Misconceptions, June to July 2021

Individuals with nonestablishment political views had a higher likelihood of COVID-19 vaccine refusal compared with those with proestablishment views in adults aged 18 years and older (adjusted incidence rate ratio, 3.26 [95% CI, 1.63-6.51]) and adults aged 60 years and older (adjusted incidence rate ratio, 2.10 [95% CI, 1.15-3.83]) after controlling for sociodemographic characteristics (eTable 5 in Supplement 1). Levels of trust in vaccine information sources, vaccine confidence, and vaccine misconceptions also varied across stratum of political views (Figure 3). Political participation assessed during the 2014 Occupy Central protests (waves 3-4) was also associated with COVID-19 vaccine refusal (eFigure 4, eTable 6, and eTable 7 in Supplement 1).

#### Trust in Vaccine Information Sources

**Figure 4.**
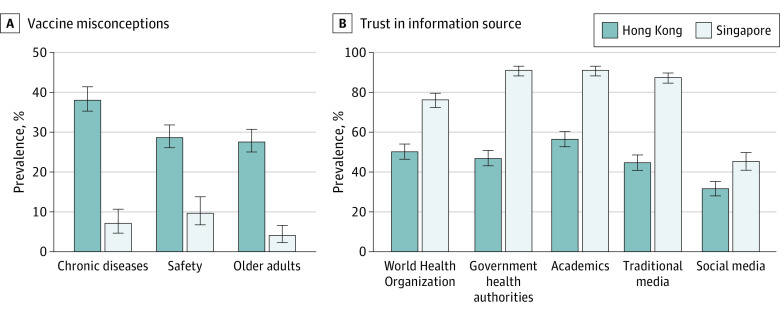
COVID-19 Vaccine Misconceptions and Trust in Information Sources in Hong Kong (June to July 2021) and Singapore (May 2020 to June 2021)

In Hong Kong, participants reported that physicians (69.3% [95% CI, 65.6%-72.8%] of participants) and academics (56.5% [95% CI, 52.7%-60.3%] of participants) were the most trusted information sources for COVID-19 vaccines, followed by the WHO (50.2% [95% CI, 46.4%-54.1%] of participants), government health authorities (47.0% [95% CI, 43.2%-50.8%] of participants), traditional media (44.7% [95% CI, 40.9%-48.5%] of participants), and social media (31.5% [95% CI, 28.0%-35.3%] of participants) (Figure 4B; eFigure 6 and eFigure 7 in Supplement 1). In Singapore, participants reported that academics (91.1% [95% CI, 88.3%-93.2%] of participants) and government health authorities (91.0% [95% CI, 88.4%-93.1%] of participants) were the most trusted information sources for COVID-19, followed by traditional media (87.4% [95% CI, 84.6%-89.8%] of participants), the WHO (76.3% [95% CI, 72.3%-79.8%] of participants), and social media (45.3% [95% CI, 40.9%-49.7%] of participants). Distrust in health authorities was associated with low vaccine confidence, vaccine misconceptions, and vaccine refusal that was independent of sociodemographic characteristics and political views (eTable 5, eTable 8, and eTable 9 in Supplement 1).

#### Vaccine Confidence

Vaccine confidence was highest before the COVID-19 vaccine rollout but declined to its lowest level during the launch of the vaccination program and widespread reports of AEFIs (eFigure 8 in Supplement 1). Subsequently, it took more than a year for vaccine confidence to recover. Low vaccine confidence was associated with vaccine refusal, adjusting for sociodemographic characteristics and political views (eTable 5 in Supplement 1).

#### Vaccine Misconceptions

More than half of the adult population (58.6% of adults) in Hong Kong reported a major misconception about COVID-19 vaccines. By contrast, 16.6% of adults in Singapore reported a major misconception (Figure 4A). In Hong Kong, 56.7% (95% CI, 53.2-60.2%) of participants opposed vaccination in adults aged 80 years and older, the age group that accounted for most COVID-19 deaths (eFigure 9 in Supplement 1).^20^ The association between misconceptions and vaccine refusal also remained after adjusting for sociodemographic characteristics and political views (eTable 5 in Supplement 1). The top 3 sources of misconceptions regarding priority groups (eg, older people or people with chronic diseases) were social media, family and friends, and physicians (eTable 10 and eTable 11 in Supplement 1).

#### Mediation Analyses for COVID-19 Vaccine Refusal

**Figure 5.**
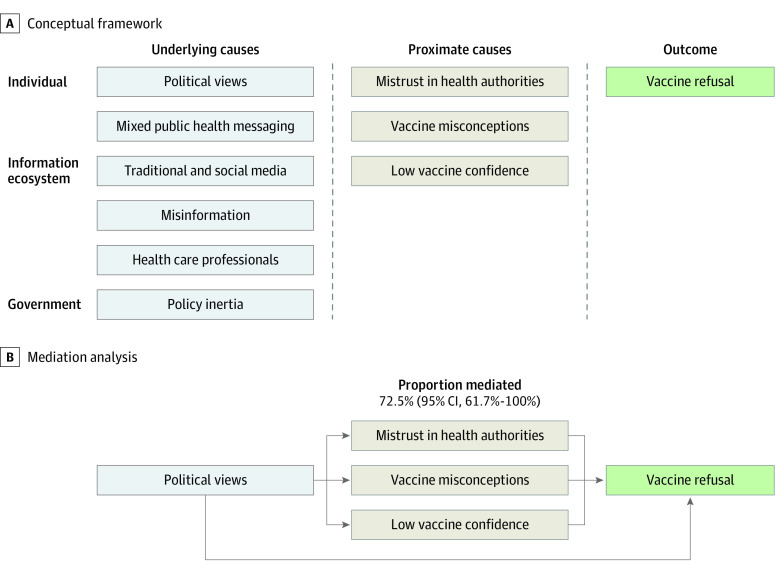
Conceptual Framework and Mediation Analysis of COVID-19 Vaccine Refusal

In exploratory analyses, we found that 72.5% (95% CI, 61.7%-100.0%) of the association between political views and vaccine refusal was via mistrust in health authorities, vaccine misconceptions, and vaccine confidence (Figure 5B). As such, the direct association of political views with vaccine refusal was no longer significant (eTable 9 in Supplement 1).

### Potential Gains in Vaccination Willingness

#### Population Attributable Fractions for COVID-19 Vaccine Refusal

Vaccine refusal in adults aged 60 years and older was attributable to low vaccine confidence with regards to importance (36.3% [95% CI, 19.7%-51.6%] of participants), safety (24.6% [95% CI, 10.4%-38.8%] of participants) and effectiveness (18.6% [95% CI, 6.0%-31.4%] of participants) of vaccines; distrust in the WHO (25.3% [95% CI, 9.5%-42.4%] of participants), government health authorities (21.5% [95% CI, 4.4%-39.0%] of participants), and academics (19.6% [95% CI, 4.5%-34.5%] of participants); and political views (17.7% [95% CI, 3.1%-32.5%] of participants) (eFigure 10 in Supplement 1). When modeled simultaneously, the joint population attributable fraction of the 4 determinants (ie, mistrust in health authorities, low vaccine confidence, vaccine misconceptions, and political views) was estimated to be 75.0% (95% CI, 59.1%-90.9%) of vaccine refusal in adults aged 18 years and older, 82.2% (95% CI, 62.3%-100.0%) of vaccine refusal in adults aged 18 to 59 years, and 69.3% ([95% CI, 47.2%-91.4%) of vaccine refusal in adults aged 60 years and older (eFigure 11 in Supplement 1).

#### Potential Gains in Vaccination Willingness

We examined the absolute changes in vaccine refusal in November 2021 (ie, before the Omicron variant began to impact Hong Kong) by counterfactual scenarios (eFigure 10 in Supplement 1). In adults aged 60 years and older, building confidence in the COVID-19 vaccine could be associated with absolute decreases in vaccine refusal by 10.2 (95% CI, 5.6-14.8) percentage points for vaccine importance, 6.9 (95% CI, 3.0-11.2) percentage points for vaccine safety, and 5.2 (95% CI, 1.7-9.1) percentage points for vaccine effectiveness. If mistrust were addressed, this could be associated with an absolute decrease in vaccine refusal in 7.1 (95% CI, 2.7-11.9) percentage points for trust in the WHO, 6.1 (95% CI, 1.2-11.0) percentage points for trust in government health authorities, and 5.5 (95% CI, 1.3-9.9) percentage points for trust in academics.

## Discussion

This cohort study assessed the evolution of vaccination willingness from before vaccine rollout to mass vaccination using data from a prospective population-based study. We demonstrated that political views may exert substantial short- and long-term associations with COVID-19 vaccine refusal. However, changing political beliefs may not be feasible, and such attempts could further undermine trust in government and health authorities.^24,25,26,27^ Nevertheless, we showed that the association of political views with vaccine refusal could be largely mediated (72.5%) by modifiable mediators (ie, mistrust in health authorities, low vaccine confidence, and vaccine misconceptions). This finding should be considered as exploratory, and if verified, it would have important implications that the impact of political views on vaccine refusal can be mitigated.^15,24,28,29^

More than half of the Hong Kong adult population (58.6%) held at least 1 misconception regarding COVID-19 vaccines, while the corresponding figure was 16.6% for Singapore, which launched one of the most successful vaccination programs worldwide.^2^ However, dispelling misconceptions can be difficult, since even brief exposure to misinformation could be entrenched into an individual’s long-term memory and the public’s consciousness.^29^ Indeed, with widespread misinformation, 1 in 4 adults in Hong Kong believed that COVID-19 vaccines were more harmful than the infection itself. This misconception remained for 1 year, even when Hong Kong recorded the world’s highest daily COVID-19 mortality.^2^

A natural candidate to prevent the onset of vaccine misconceptions could be engendering trust in health authorities (eg, the WHO, government health authorities, and academics), as trust in health authorities could confer resilience against misinformation and misconceptions while promoting vaccine confidence and uptake.^25,28^ As such, trust could be fundamental to attaining good vaccination coverage as well as explaining Hong Kong’s high COVID-19 mortality.^24,25^ Indeed, the mistrust in government health authorities sets Hong Kong apart from other places that implemented COVID-19 elimination strategies.^6,30^ Less than half (47.0%) of Hong Kong adults trusted their government health authorities on COVID-19 vaccines. By contrast, 91.0% of adults in Singapore trusted their government health authorities during the pandemic.

When vaccine refusal is widespread in a population, systemic underlying causes, rather than individual characteristics, are more likely explanations.^31,32,33^ Here, we identified 4 factors that could explain 75.0% of vaccine refusal in adults. Political views were an underlying factor associated with vaccine refusal; however, they were not the root cause of all causal pathways. Notably, the associations of mistrust, vaccine confidence, and misconceptions with vaccine refusal were independent of political views. This indicates that the underlying determinants of vaccine refusal go beyond political views.^34^ To attain sufficient vaccination coverage and protect population health, a multipronged approach to address individual and underlying causes of vaccine refusal should be adopted.^25,35^

A number of underlying factors associated with vaccine refusal bear mention. First, public health messaging was mixed.^36^ For transparency, health authorities in Hong Kong announced all serious AEFIs. However, the causality between COVID-19 vaccines and AEFIs was not made clear to the public at times.^37,38^ As such, AEFIs were widely reported in the media, and vaccine misconceptions were pervasive in the public.^33^ Second, a number of public policies that aimed to enhance vaccine uptake (eg, workplace vaccine mandates) were not applicable to older adults. This may be why approximately three-quarters of residential care home residents were unvaccinated when the Omicron variant began to appear in Hong Kong, and this accounted for most COVID-19 deaths.^20,39^ Therefore, the effectiveness of vaccine policies in high-risk groups needs to be considered.^40^

Third, trusted messengers are needed to allay public anxiety over new vaccines.^25,28,41^ This includes health care professionals (HCPs), who play a crucial role in influencing vaccine decision-making.^42,43,44^ Therefore, vaccine hesitancy among HCPs could undermine efforts to promote vaccine uptake.^45^ Indeed, our findings suggest that physicians, the most trusted source of health information in Hong Kong, were also a significant source of vaccine misconceptions in Hong Kong.^46^ This may be due to many physicians being unvaccinated themselves.^47^ Only 35% of HCPs in Hong Kong’s public sector received COVID-19 vaccines during the initial rollout, compared with more than 90% of their counterparts in Mainland China and England.^48,49,50^ Vaccine refusal even among HCPs reaffirms that the drivers of vaccine refusal transcend individual characteristics, such as scientific literacy, and the importance of targeting the systemic causes of vaccine hesistancy.^14^ Promoting vaccine confidence in health care professionals should be prioritized, given their substantial influence, and their support is vital for building trust in government health agencies and the WHO.^24,42,43^

### Limitations

Our study has several limitations. First, as with other long-running cohorts, there could be potential attrition bias. Nevertheless, the application of censoring weights and raking has enhanced the representativeness of our sample.^51,52^ Second, the associations of mistrust and political views with vaccine refusal could be susceptible to reverse causality. Vaccine mandates issued by the government could lead to mistrust in health authorities and negative views toward the government among unvaccinated individuals.^53,54,55^ However, we assessed mistrust prior to the announcements of vaccine mandates or passes. Moreover, we prospectively assessed political views during the 2014 Occupy Central protests and the 2019 to 2020 social unrest, and both yielded consistent results with vaccine refusal. Third, as with all analyses using population attributable fraction, causality is assumed between the exposure and the outcome. Nonetheless, assumptions in our study are empirically supported and the longitudinal design could help improve causal inference.^6,15,24,29^ Fourth, in the context of multiple associated factors, attributable risk for an individual factor without adjustment of other factors could result in an overestimation.^56^ However, we also calculated sequential and average attributable fractions, which controls for all other measured factors, and reported the joint contributions of determinants to vaccine refusal. Fifth, the generalizability of our findings to other populations would need to be evaluated. Sixth, we could not include all potential determinants of vaccine refusal. Nonetheless, the factors assessed could account for up to 75.0% of vaccine refusal, suggesting that key determinants of vaccine refusal have been included.

## Conclusions

This cohort study was the largest and most comprehensive assessment of COVID-19 vaccine refusal in Hong Kong to our knowledge, and we found that the factors associated with Hong Kong’s poor vaccination coverage could be modifiable. This serves as a cautionary tale, given the perpetual challenge of emerging infectious diseases.^54,57^ Here, we provide evidence that suggests that trust in health authorities is fundamental to overcoming vaccine refusal.^25^ Engendering trust could benefit from a whole-of-society approach, which includes a shift from a single stakeholder to a coordinated effort among HCPs, scientists, local organizations, public health agencies, and others.^58^ In particular, trusted messengers should be engaged in community outreach, building public trust, and promoting vaccine confidence.^28,41^ Finally, governments need to prioritize the credibility of public health agencies, such as grounding health policies and messaging in science.^26,59^ This could help public health agencies improve vaccination coverage and protect population health even when society is divided.^25,31,54^

## Notes

Supplement 1.
eMethods.
**eAppendix.** Supplementary Results **eFigure 1.** COVID-19 Mortality in Settings That Implemented the Elimination Policy in the Asia-Pacific **eFigure 2.** COVID-19 Vaccine Uptake in Settings That Implemented the Elimination Policy in the Asia-Pacific **eFigure 3.** Sampling and Retention of Participants in 20 Waves of Longitudinal Data in a Population-Based Cohort, 2009-2022 **eFigure 4.** Vaccination Willingness and Trust in COVID-19 Vaccine Information Sources in Hong Kong by Participation in the 2014 Occupy Central **eFigure 5.** Residual Check of Selected ARIMAX Model for Interrupted Time-Series Analyses **eFigure 6.** Trends in Vaccination Willingness in Older Adults (≥60 Years), 2020-2022 **eFigure 7.** Trends in Trust in Vaccine Information Sources in Hong Kong, 2021-2022 **eFigure 8.** Trends in COVID-19 Vaccine Confidence in Hong Kong, 2020-2022 **eFigure 9.** Trends in Vaccine Misconceptions in Hong Kong, 2021-2022 **eFigure 10.** Population Attributable Fractions for Factors, Assessed in June-July 2021, Associated With Vaccine Refusal and Absolute Reduction of Vaccine Refusal in November 2021 **eFigure 11.** Population Attributable Fractions for Factors, Assessed in June-July 2021, Associated With Vaccine Refusal and Absolute Reduction of Vaccine Refusal in November 2021 **eTable 1.** COVID-19 Vaccine Uptake Among Older Adults Before the First Documented Local Omicron Transmission **eTable 2.** List of Outcomes and Exposures Used in Hong Kong **eTable 3.** Demographic Composition of Wave 20 Compared to 2016 Population By-Census of Hong Kong **eTable 4.** Interrupted Time-Series Analyses of Intervention Measures and Daily COVID-19 Vaccination Appointments **eTable 5.** Associations of Political Views, Trust in Information Sources, Vaccine Misconceptions, and Vaccine Confidence in June-July 2021, With Vaccine Refusal in the General Population (≥18 years) and Older Adults (≥60 years) in November 2021 **eTable 6.** Associations of Political Views During 2014 Occupy Central With Vaccine Refusal Over COVID-19 Pandemic **eTable 7.** Association of Political Views During 2019 Social Unrest With Trust in COVID-19 Vaccine Information Sources, Vaccine Misconceptions, and Vaccine Confidence in June 2021 **eTable 8.** Association Between Trust in COVID-19 Vaccine Information Sources in June-July 2021, and Endorsement of Vaccine Misconceptions and COVID-19 Vaccine Confidence in November 2021 **eTable 9.** Direct and Indirect Effects of Political Views on Vaccine Refusal in November 2021 Via Mistrust in Vaccine Information Sources, Vaccine Misconceptions and Vaccine Confidence in June-July 2021 **eTable 10.** Social Influences for Decision-Making on COVID-19 Vaccine Uptake Among Older Adults, February 2022 **eTable 11.** Information Source for Misconceptions Regarding Priority Groups for COVID-19 Vaccination, March 2022
eReferences.

Supplement 2.
Data Sharing Statement
