# Supplementary material for: COVID-19 Vaccination Willingness and Reasons for Vaccine Refusal
Source: JAMA Netw Open. 2023 Oct 19;6(10):e2337909. doi: 10.1001/jamanetworkopen.2023.37909 (PMC10587797; doi:10.1001/jamanetworkopen.2023.37909)
Supplement: Supplement 1. — eMethods. eAppendix. Supplementary Results eFigure 1. COVID-19 Mortality in Settings That Implemented the Elimination Policy in the Asia-Pacific eFigure 2. COVID-19 Vaccine Uptake in Settings That Implemented the Elimination Policy in the Asia-Pacific eFigure 3. Sampling and Retention of Participants in 20 Waves of Longitudinal Data in a Population-Based Cohort, 2009-2022 eFigure 4. Vaccination Willingness and Trust in COVID-19 Vaccine Information Sources in Hong Kong by Participation in the 2014 Occupy Central eFigure 5. Residual Check of Selected ARIMAX Model for Interrupted Time-Series Analyses eFigure 6. Trends in Vaccination Willingness in Older Adults (≥60 Years), 2020-2022 eFigure 7. Trends in Trust in Vaccine Information Sources in Hong Kong, 2021-2022 eFigure 8. Trends in COVID-19 Vaccine Confidence in Hong Kong, 2020-2022 eFigure 9. Trends in Vaccine Misconceptions in Hong Kong, 2021-2022 eFigure 10. Population Attributable Fractions for Factors, Assessed in June-July 2021, Associated With Vaccine Refusal and Absolute Reduction of Vaccine Refusal in November 2021 eFigure 11. Population Attributable Fractions for Factors, Assessed in June-July 2021, Associated With Vaccine Refusal and Absolute Reduction of Vaccine Refusal in November 2021 eTable 1. COVID-19 Vaccine Uptake Among Older Adults Before the First Documented Local Omicron Transmission eTable 2. List of Outcomes and Exposures Used in Hong Kong eTable 3. Demographic Composition of Wave 20 Compared to 2016 Population By-Census of Hong Kong eTable 4. Interrupted Time-Series Analyses of Intervention Measures and Daily COVID-19 Vaccination Appointments eTable 5. Associations of Political Views, Trust in Information Sources, Vaccine Misconceptions, and Vaccine Confidence in June-July 2021, With Vaccine Refusal in the General Population (≥18 years) and Older Adults (≥60 years) in November 2021 eTable 6. Associations of Political Views During 2014 Occupy Central With Vaccine Refusal Over COVID-19 Pandemic [file jamanetwopen-e2337909-s001.pdf]

Supplemental Online Content

Lun P, Ning K, Wang Y, et al. COVID-19 vaccination willingness and reasons for vaccine refusal. *JAMA Netw Open*. 2023;6(10):e2337909.
doi:10.1001/jamanetworkopen.2023.37909

eMethods.
eAppendix. Supplementary Results
eFigure 1. COVID-19 Mortality in Settings That Implemented the Elimination Policy in the Asia-Pacific
eFigure 2. COVID-19 Vaccine Uptake in Settings That Implemented the Elimination Policy in the Asia-Pacific
eFigure 3. Sampling and Retention of Participants in 20 Waves of Longitudinal Data in a Population-Based Cohort, 2009-2022
eFigure 4. Vaccination Willingness and Trust in COVID-19 Vaccine Information Sources in Hong Kong by Participation in the 2014 Occupy Central
eFigure 5. Residual Check of Selected ARIMAX Model for Interrupted Time-Series Analyses
eFigure 6. Trends in Vaccination Willingness in Older Adults (≥60 Years), 2020-2022
eFigure 7. Trends in Trust in Vaccine Information Sources in Hong Kong, 2021-2022
eFigure 8. Trends in COVID-19 Vaccine Confidence in Hong Kong, 2020-2022
eFigure 9. Trends in Vaccine Misconceptions in Hong Kong, 2021-2022
eFigure 10. Population Attributable Fractions for Factors, Assessed in June-July 2021, Associated With Vaccine Refusal and Absolute Reduction of Vaccine Refusal in November 2021
eFigure 11. Population Attributable Fractions for Factors, Assessed in June-July 2021, Associated With Vaccine Refusal and Absolute Reduction of Vaccine Refusal in November 2021
eTable 1. COVID-19 Vaccine Uptake Among Older Adults Before the First Documented Local Omicron Transmission
eTable 2. List of Outcomes and Exposures Used in Hong Kong
eTable 3. Demographic Composition of Wave 20 Compared to 2016 Population By-Census of Hong Kong
eTable 4. Interrupted Time-Series Analyses of Intervention Measures and Daily COVID-19 Vaccination Appointments
eTable 5. Associations of Political Views, Trust in Information Sources, Vaccine Misconceptions, and Vaccine Confidence in June-July 2021, With Vaccine Refusal in the General Population (≥18 years) and Older Adults (≥60 years) in November 2021
eTable 6. Associations of Political Views During 2014 Occupy Central With Vaccine Refusal Over COVID-19 Pandemic
eTable 7. Association of Political Views During 2019 Social Unrest With Trust in COVID-19 Vaccine Information Sources, Vaccine Misconceptions, and Vaccine Confidence in June 2021
eTable 8. Association Between Trust in COVID-19 Vaccine Information Sources in June-July 2021 and Endorsement of Vaccine Misconceptions and COVID-19 Vaccine Confidence in November 2021
eTable 9. Direct and Indirect Effects of Political Views on Vaccine Refusal in November 2021 Via Mistrust in Vaccine Information Sources, Vaccine Misconceptions and Vaccine Confidence in June-July 2021
eTable 10. Social Influences for Decision-Making on COVID-19 Vaccine Uptake Among Older Adults, February 2022
eTable 11. Information Source for Misconceptions Regarding Priority Groups for COVID-19 Vaccination, March 2022
eReferences.

This supplemental material has been provided by the authors to give readers additional information about their work.

© 2023 Lun P et al. *JAMA Network Open.*

eMethods

Study design and participants in Singapore.

Eligible participants from the second Singapore Mental Health Study were interviewed via Zoom or in-person[1]. We used the baseline sample (n=1,129), which was enrolled from May 14th, 2020 to June 31st, 2021, and a follow-up (n=500) from October 21st, 2021 to January 16th, 2022. Written informed consent was obtained from all the participants.

Response and cooperation rates.

Response rate is the number of completed interviews divided by the number of eligible study units. Cooperation rate is the number of completed interviews divided by the number of eligible study units where contact was made[2].

Assessment of exposures.

Participants in Hong Kong reported their trust in COVID-19 vaccine information sources (i.e. World Health Organization (WHO), government health authorities, physicians, academics, and traditional and social media platforms) on a scale of 1–10 (1=complete mistrust, 10=complete trust) in waves 15–20. A score of ≤5 was considered as mistrust.

Participants in Singapore reported their trust in COVID-19 information sources (i.e. WHO, government health authorities, academics, and traditional and social media platforms) on a scale of 1–10 (1=complete mistrust, 10=complete trust) in the baseline (May 2020 to June 2021). A score of ≤5 was considered as mistrust.

The three major misconceptions about COVID-19 vaccines were assessed using the following statements in Hong Kong: "Older individuals have a greater need for COVID-19 vaccination", "Individuals with more chronic diseases have a greater need for COVID-19 vaccination", and "COVID-19 vaccines are more harmful than COVID-19 infection" in waves 15–20. Disagreement with the first two statements and agreement with the last statement were considered as vaccine misconceptions. Opposition to COVID-19 vaccination in adults aged ≥80 years was assessed in waves 17–20. We further assessed the primary information source for these beliefs in wave 19. We also assessed if participants opposed COVID-19 vaccination for adults aged ≥80 years (waves 17–20).

The three major misconceptions about COVID-19 vaccines were assessed using the following statements in Singapore: "Individuals aged 60 years and above should receive COVID-19 vaccines", "Individuals with more chronic diseases have a greater need for COVID-19 vaccines", and "COVID-19 vaccines are more harmful than COVID-19 infection" in the follow-up (October 2021 to January 2022). Disagreement with the first two statements and agreement with the last statement were considered as vaccine misconceptions.

COVID-19 vaccine confidence was assessed in Hong Kong (waves 11–18, 20) with statements adapted from the Vaccine Confidence Index on a 5-point Likert scale ranging from strongly disagree to strongly agree: "I think COVID-19 vaccines are effective", "I think COVID-19 vaccines are safe", and "I think COVID-19 vaccines are important for children to have"[3]. Strongly agree and agree were grouped together versus the other options[4].

COVID-19 public policies on vaccine uptake included 1) lottery-based incentives (e.g. residential apartment) announced from May 26th, 2021; 2) vaccine mandates for civil servants and staff at schools, care homes, and public hospitals announced on August 2nd, 2021; 3) vaccine pass (restricting access to premises such as restaurants, public buildings, and leisure facilities) announced on December 31st, 2021 and enacted on February 24th, 2022, and 4) reopening of premises under the vaccine pass announced on April 14th, 2022 and enacted on April 21st, 2022[5-9].

Assessment of co-variables.

Media reports on Adverse Events Following COVID-19 Immunisation (AEFIs) were extracted from WiseNews, a dataset of news clippings from Hong Kong traditional newspaper publishers and online news media, between August 1st, 2020 and May 30th, 2022[10]. Examples of AEFIs recognised by Drug Office, Department of Health, of the Government of Hong Kong Special Administration Region include anaphylaxis, Bell's palsy, and encephalomyelitis[11]. The news articles must include the words "COVID" and "vaccines(s)". The articles must also include any of the following words or their Chinese equivalents: "side

© 2023 Lun P et al. *JAMA Network Open.*

effects", "adverse events", "Advisory Panel on COVID-19 Vaccines", "Expert Committee on Clinical Events Assessment", "loss of function", "anaphylaxis", "anaphylactoid reaction", "acute peripheral facial paralysis", "Bell's palsy", "encephalopathy", "myocarditis", "inflammation of the heart muscle", "pericarditis", "inflammation of the lining outside the heart", "thrombocytopenia", "thrombosis with thrombocytopenia syndrome", and "transverse myelitis".

Post-stratification weighting and raking and calculation of Cohen's w.

Post-stratification weighting was estimated by calculating censoring weights. For Hong Kong, it was defined as the inverse probability of participating in the study after wave 2 and estimated using logistic regression with sociodemographic characteristics at wave 2 (i.e. age, sex, education attainment, marital status, employment status, household income and housing type). Raking was then applied for the sample to be representative of the 2016 Population By-census of Hong Kong using age, sex, education attainment, marital status, monthly household income and housing type.

For Singapore, it was defined as the inverse probability of participating in the follow-up after the baseline and estimated using logistic regression with sociodemographic characteristics at baseline (i.e. age, sex, education attainment, marital status and ethnicity). Raking was then applied for the baseline sample to be representative of the 2020 Population By-census of Singapore, and the follow-up sample to be representative of the 2021 population statistics from the Singapore Department of Statistics using age, sex, education attainment, marital status, and ethnicity.

Cohen's w is a measure to assess association between two nominal variables, and is equivalent to the effect size for a chi-square test of association[12]. We calculated Cohen's w using the "ES.w1" function from the "pwr" package in R to compare the distribution of each sociodemographic between our sample and the 2016 Population By-census of Hong Kong. The effect size measured by Cohen's w is considered small for values close to 0.1, medium for around 0.3, large for around 0.5.

Interrupted time-series analyses.

We obtained the official daily number of COVID-19 vaccination appointments in Hong Kong from February 23rd, 2021 to May 30th, 2022 to assess the impact of public policies and interventions on vaccine uptake. These included: 1) lottery-based incentives (e.g. residential apartment) announced from May 26th, 2021; 2) vaccine mandates for civil servants and staff at schools, care homes, and public hospitals announced on August 2nd, 2021; 3) vaccine pass (restricting access to premises such as restaurants, public buildings, and leisure facilities) announced on December 31st, 2021 and enacted on February 24th, 2022, and 4) reopening of premises under the vaccine pass announced on April 14th, 2022 and enacted on April 21st, 2022[5-9]. These measures were examined in the interrupted time-series analyses to examine their impact on daily COVID-19 vaccination appointments. Given the high public awareness of lottery-based incentives, a new equilibrium was assumed to reach soon after them being announced and sustained up till the next measure (i.e. workplace vaccine mandates). We therefore used step functions to model each of the four interventions. In addition, Lunar New Year (January 31st–February 2nd, 2022) and surge of Omicron wave (February 7th, 2022–April 14th, 2022) were taken into account.

Autoregressive Integrated Moving Average Exogenous Variable Model (ARIMAX) was used to incorporate exogenous covariates (i.e. the four interventions, Lunar New Year and surge of Omicron wave) into the time series model, and daily vaccination appointments were log transformed to stabilise the variance of time series[13]. Due to model parsimony and the lack of autocorrelation in the residuals, we selected an ARIMAX model of (0,1,2)*(0,1,1) to fit the data. Percent change in the vaccine appointment numbers after the implementation of any intervention was estimated by (exponentiate of the coefficient-1)*100.

Generalised estimating equations between political views in 2014 and COVID-19 vaccine refusal.

We used generalised estimating equations with an independent correlation matrix to examine the association between political views during the 2014 Occupy Central (waves 3–4) and COVID-19 vaccine refusal (waves 11–17).

Causal mediation analyses.

We conducted causal mediation analyses using the CMAverse packages in R[14]. The proposed causal diagram for the relationships between political views, trust in vaccine information sources, vaccine misconceptions, vaccination confidence, and vaccine refusal is shown in Fig. 5, with sociodemographics (i.e. age, sex, education attainment, marital status, employment status and monthly household income) as baseline confounders. Exposure, mediators, and outcome were all dichotomised: political views (non-

© 2023 Lun P et al. *JAMA Network Open.*

establishment view and others); trust in COVID-19 vaccine information sources (mistrust and trust); vaccine misconceptions (yes and no); vaccine confidence (not agree and agree); and vaccine refusal (yes and no).

Mediator and outcome were modelled using Poisson distribution with log link function. A regression-based approach was adopted, and natural direct and indirect effects were estimated through direct counterfactual imputation estimation. Multiple imputation was performed to handle missing values (number of imputed dataset=20). Standard errors of causal effects were estimated through bootstrapping (number of bootstrapping=200). In interpreting our results, assumptions included no unmeasured confounding between exposure and outcome, mediators and outcome, and exposure and mediators after adjusting for sociodemographics; no intermediate confounding between mediators and outcome induced by exposure; no interactions between exposure and mediators; no measurement error; correct parametric specification of the models; no interference, and causal consistency[14,15].

We examined the indirect effects of political views on vaccine refusal via mistrust in vaccine information sources, vaccine misconceptions, and low vaccine confidence in tandem (eTable 8). Proportion mediated was reported. We also calculated adjusted incidence rate ratios for the exposure–mediator, and mediator–outcome relationships in each mediation model. Adjusted incidence rate ratios were calculated by extracting the coefficients and standard errors from mediation models built for each imputed dataset that were stored in CMAverse and combining them using Rubin's rule[16].

Estimation of population attributable fractions of vaccine refusal and absolute reduction in vaccine refusal if a risk factor was eliminated.

We examined the determinants of vaccine refusal at wave 17 as this timepoint preceded Hong Kong's major surge in COVID-19 mortality. Although vaccinations increased during the Omicron surge (waves 18–19), it was often too late for older adults receiving their first dose to be protected against severe disease or death during the Omicron wave[17-19]. We estimated the sequential and average population attributable fractions (PAFs) of vaccine refusal for each determinant as well as their joint contribution, utilising the "averisk" packages in R[20,21]. Vaccine refusal was modelled using logistic regression, and the determinants were modelled simultaneously (i.e. political views, trust in COVID-19 vaccine information sources from WHO, government health authorities and academics, COVID-19 vaccine misconceptions about older adults, chronic diseases and safety, COVID-19 vaccine confidence in its effectiveness, safety and importance for children to have) while adjusting for sociodemographics. To handle the missing data, 20 datasets were imputed. Point estimates and standard errors of each imputed dataset were extracted from the "averisk" package and combined using Rubin's rule[16].

To estimate the individual contribution of determinants to vaccine refusal, we used the following estimation procedures: First, we applied robust Poisson regression to model the associations of political views during 2019 social unrest, trust in COVID-19 vaccine information sources, COVID-19 vaccine misconceptions, and COVID-19 vaccine confidence at wave 16 with vaccine refusal at wave 17 in separate models (hereinafter "$M_{Poisson}$"), adjusting for sociodemographics. Political views were additionally adjusted when modelling the association of trust in vaccine information sources, vaccine misconceptions and vaccine confidence with vaccine refusal. Second, a predicted probability (P_o) of being vaccine-hesitant was obtained for each participant under their observed level of covariables in the model. To simulate the counterfactual condition, the following were shifted: 1) political views from non-establishment to pro-establishment/neutral, 2) mistrust to trust for COVID-19 vaccine information sources, 3) vaccine misconception to no vaccine misconception, and 4) from not agreeing with statements regarding vaccine confidence to agreeing. A simulated probability (P_s) of being vaccine-hesitant under the counterfactual condition was then obtained using the same set of estimates from $M_{Poisson}$[22].

The absolute reduction in vaccine refusal in counterfactual scenarios was estimated by averaging the difference between predicted probability and simulated probability (P_o–P_s) across all participants. Population attributable fractions were calculated by averaging the fraction of the absolute reduction divided by P_o across all participants. Missing data were imputed using multiple imputation by chained equation, and results were combined from 20 imputed datasets using Rubin's rule[23]. Confidence intervals for population attributable fraction and absolute reduction of vaccine refusal were estimated following the MI Boot (Pooled Sample) algorithm[24]. Specifically, 20 datasets were imputed, and then 500 bootstraps were implemented for each imputed dataset (bootstrap for complex survey design using bs4rw package in Stata was applied to incorporate weights) and all bootstrapping results were stored[25]. Finally, 20*500 estimates were ranked and the 2.5th and 97.5th percentiles were extracted as the lower and upper limit of the confidence interval, respectively.

© 2023 Lun P et al. *JAMA Network Open.*

eResults

Long-term association between political views and COVID-19 vaccine refusal.

Political participation during the 2014 Occupy Central was also associated with COVID-19 vaccine refusal eight years later (eTable 5 and eFigure 4).

Population attributable fractions for COVID-19 vaccine refusal.

Vaccine refusal in adults aged 18–59 years was attributable to low vaccine confidence with regards to safety (60.5%, 95% CI 43.5–75.8), effectiveness (46.2%, 32.1–59.4), and importance of vaccines (40.5%, 21.0–60.3), political views (48.7%, 20.4–72.4), distrust in WHO (34.4%, 12.0–55.4), government health authorities (34.2%, 7.1–58.1), and academics (19.1%, 3.0–36.1), and vaccine misconceptions (29.9%, 1.4–52.7) (eFigure 11).

Potential gains in vaccination willingness.

In adults aged 18–59 years, building vaccine confidence with regard to the safety, effectiveness, and importance of COVID-19 vaccines could change vaccine refusal by 8.8% (6.2–11.9), 6.7% (4.6–9.2), and 5.8% (3.0–9.1), respectively. If mistrust in WHO, government health authorities, and academics were addressed, the absolute changes in vaccine refusal could be 5.0% (1.8–8.4), 4.9% (1.1–8.8), and 2.8% (0.4–5.4), respectively. If the three major vaccine misconceptions were removed, this could have reduced vaccine refusal by 4.0% (1.0–7.2) (eFigure 11). Shifting political views from non-establishment to neutral or pro-establishment may change vaccine refusal by 7.0% (2.9–11.0) and 5.0% (0.9–9.1) in adults aged 18-59 years and ≥60 years, respectively.

© 2023 Lun P et al. *JAMA Network Open.*

eFigure 1. COVID-19 mortality in settings that implemented the elimination policy in the Asia-Pacific.

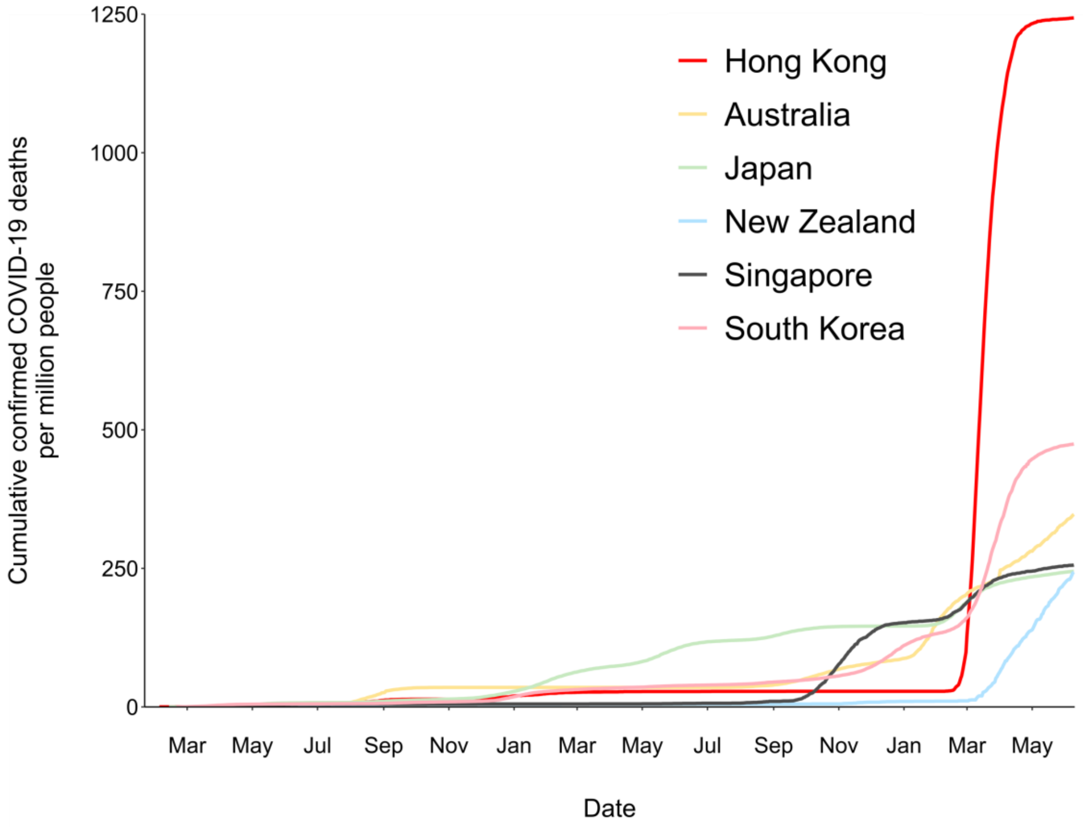

Seven-day rolling-average of daily cumulative confirmed cases of COVID-19 deaths, 2020–2022.

© 2023 Lun P et al. *JAMA Network Open.*

eFigure 2. COVID- vaccine uptake in settings that implemented the elimination policy in the Asia-Pacific.

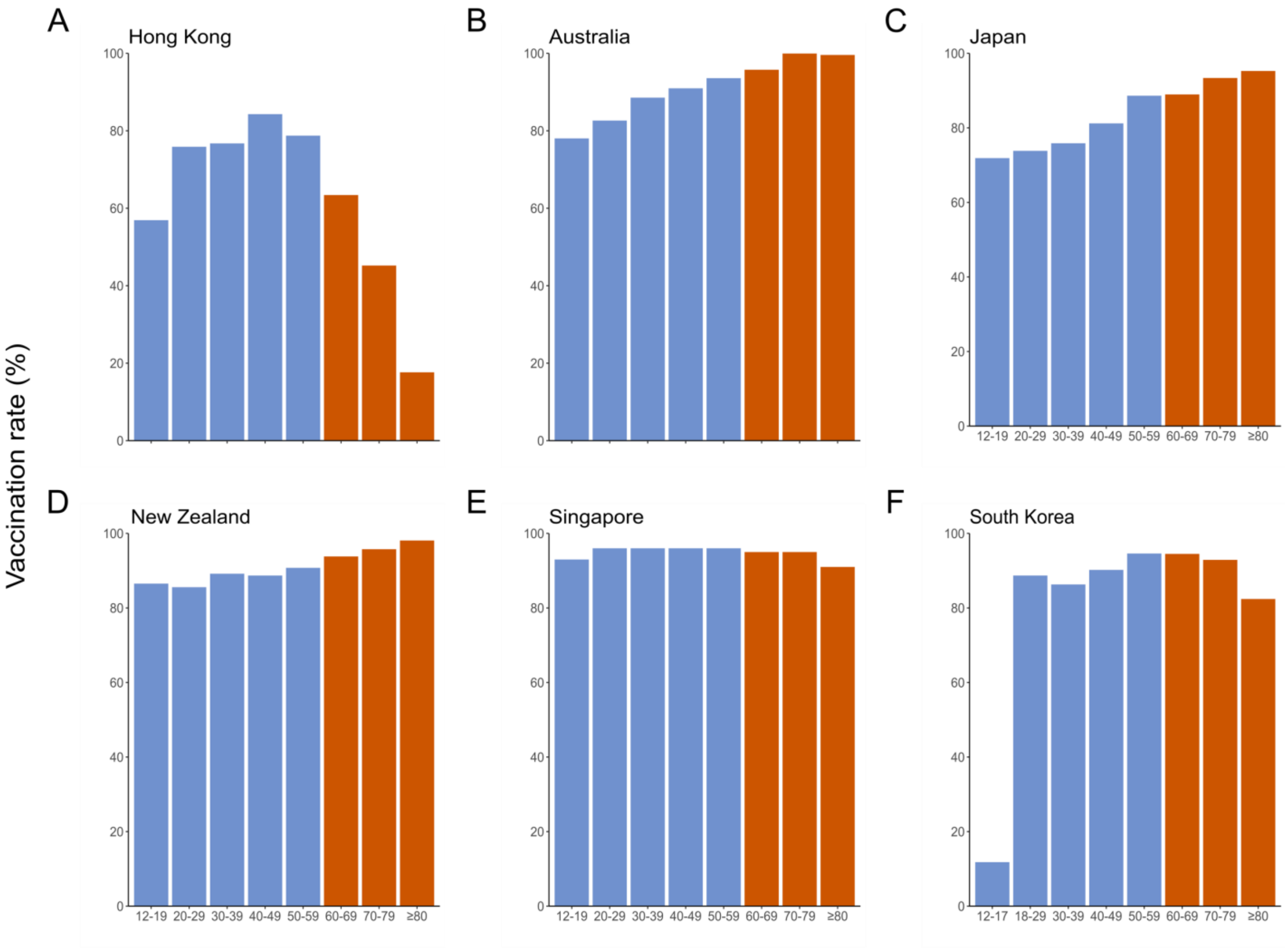

Proportion of the population that received ≥2 doses of COVID-19 vaccines 14 days before first documented local Omicron transmission.

© 2023 Lun P et al. *JAMA Network Open.*

eFigure 3. Sampling and retention of participants in 20 waves of longitudinal data in a population-based cohort, 2009–2022.

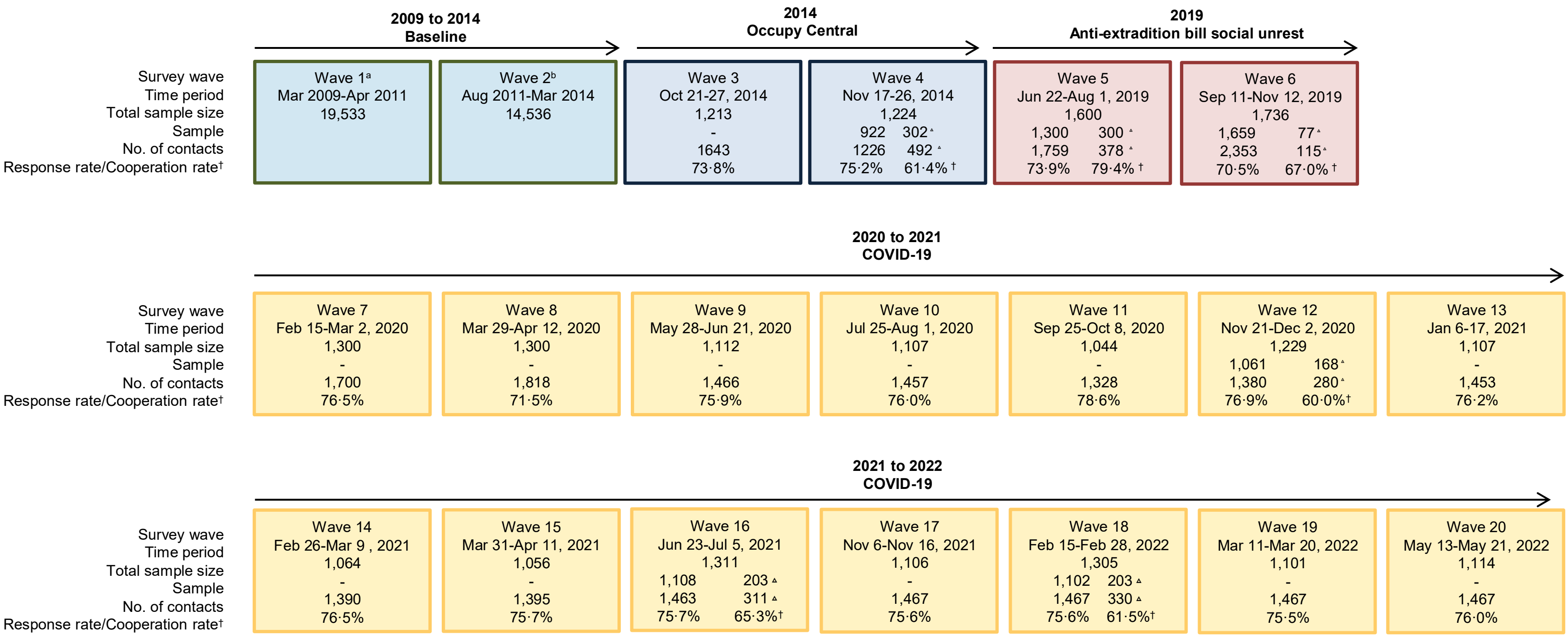

[a]Sample size of original pool (aged ≥ 10 years) in wave 1.
[b]Sample size of wave 2 sampling frame (aged ≥ 10 years).
▲ Replenishment random samples from wave 2.

© 2023 Lun P et al. *JAMA Network Open.*

eFigure 4. Vaccination willingness and trust in COVID-19 vaccine information sources in Hong Kong by participation in the 2014 Occupy Central.

A

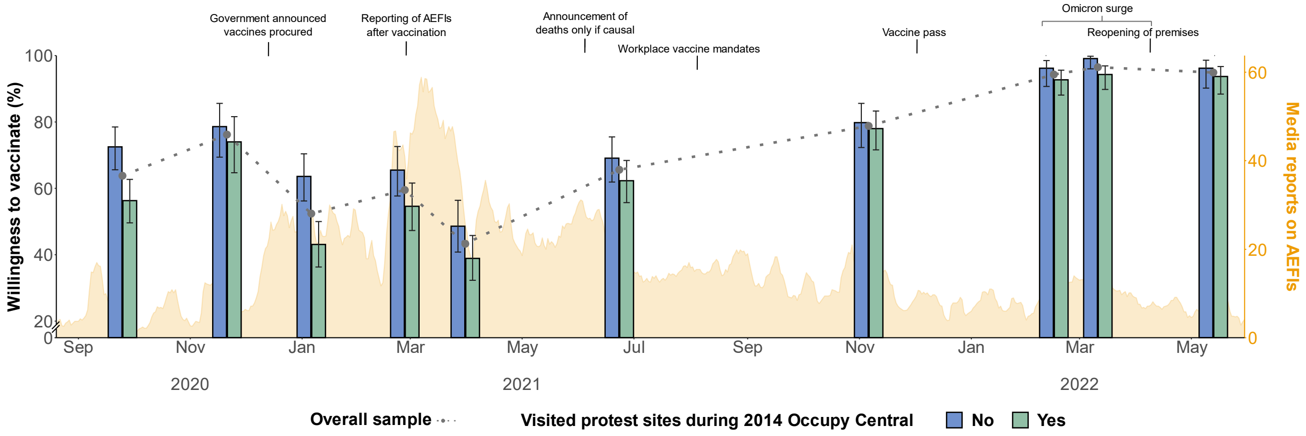

B

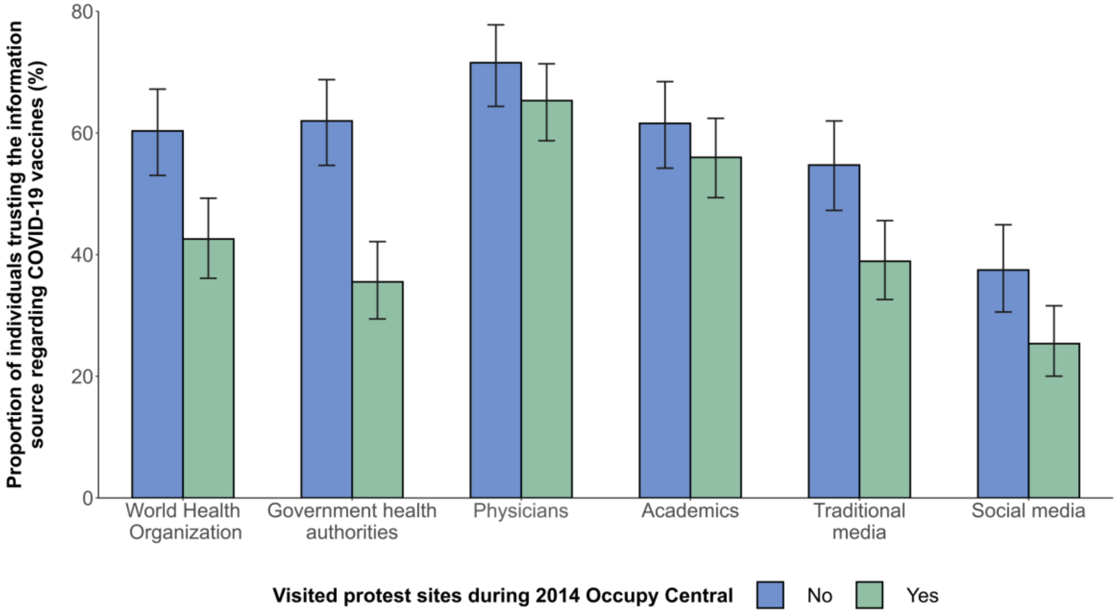

(A) Trend in vaccination willingness from 2020 to 2022, overlaid on media reports from WiseNews on Adverse Events Following COVID-19 Immunisation (AEFIs). Measures to enhance vaccine uptake included announcements of vaccine lotteries, vaccine mandates for civil servants, staff at schools, care homes, and public hospitals, implementation of vaccine pass (e.g. restricting access to premises such as restaurants, public buildings, and leisure facilities), and reopening of premises under the vaccine pass. (B) Trust in COVID-19 vaccine information sources in the adult population in June–July 2021. Error bars indicate the 95% confidence intervals. Political views were assessed in waves 3–4 during the 2014 Occupy Central. Participants who participated or visited the protest sites were classified as non-establishment. Participants that did not visit the protest sites or participate in the movement were classified as pro-establishment or neutral.

© 2023 Lun P et al. *JAMA Network Open.*

eFigure 5. Residual check of selected ARIMAX model for interrupted time-series analyses.

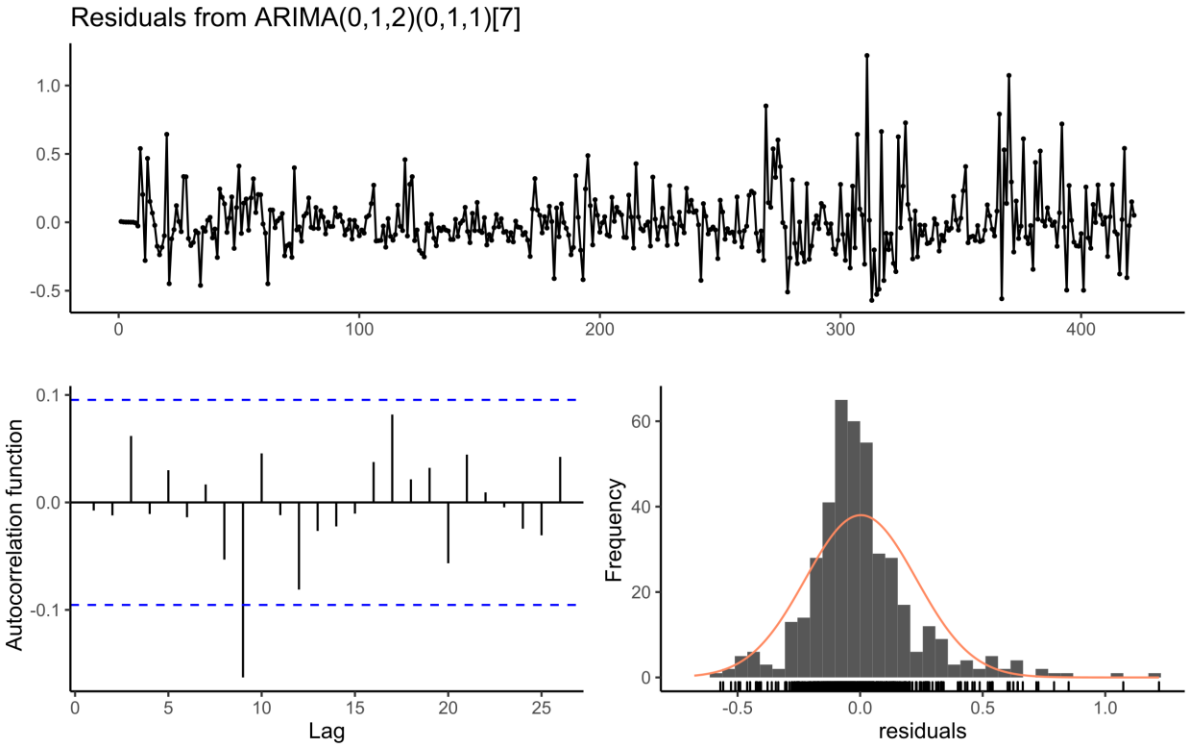

The residual plots of an ARIMAX model of (0,1,2)*(0,1,1) [7] are shown above. The upper plot indicates that the residuals were white noise with no obvious pattern. The lower left plot indicates there was no auto-correlation among lagged residuals, which was also tested by the Ljung-Box test. The lower right plot shows the distribution of residuals and compares it with the normal distribution. Those residual plots indicate an adequate fit of our data using the selected model.

© 2023 Lun P et al. *JAMA Network Open.*

eFigure 6. Trends in vaccination willingness in older adults (≥60 years), 2020–2022.

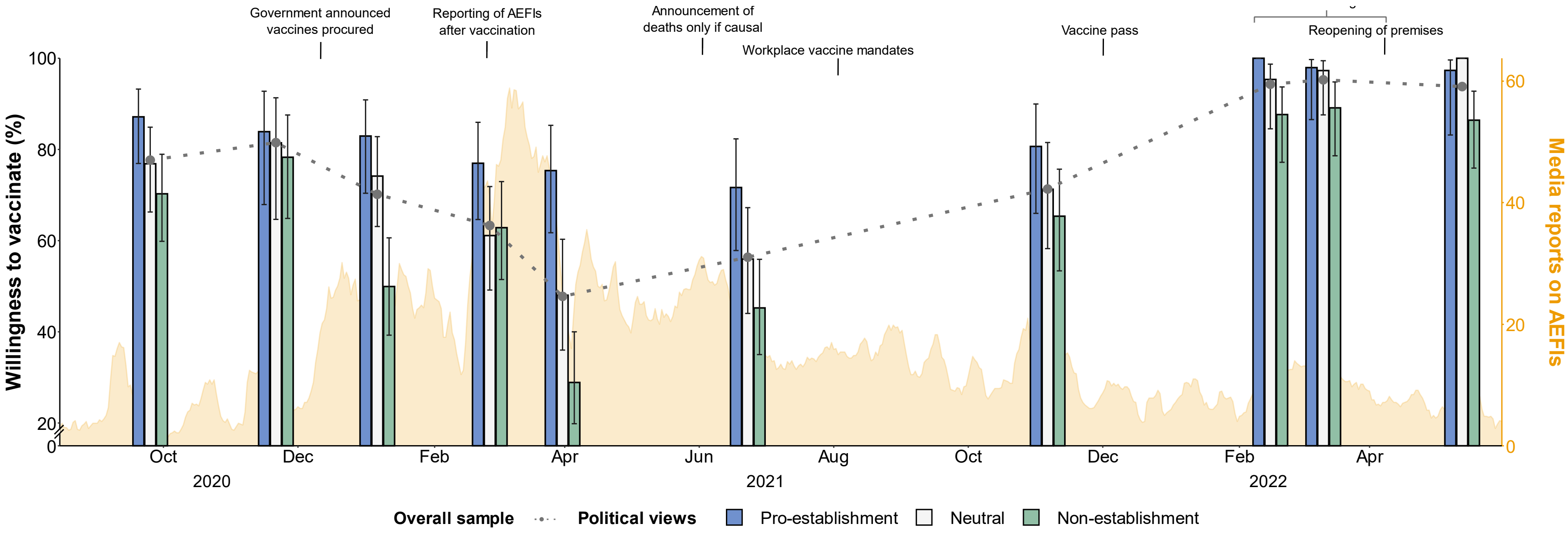

© 2023 Lun P et al. *JAMA Network Open.*

eFigure 7. Trends in trust in vaccine information sources in Hong Kong, 2021–2022.

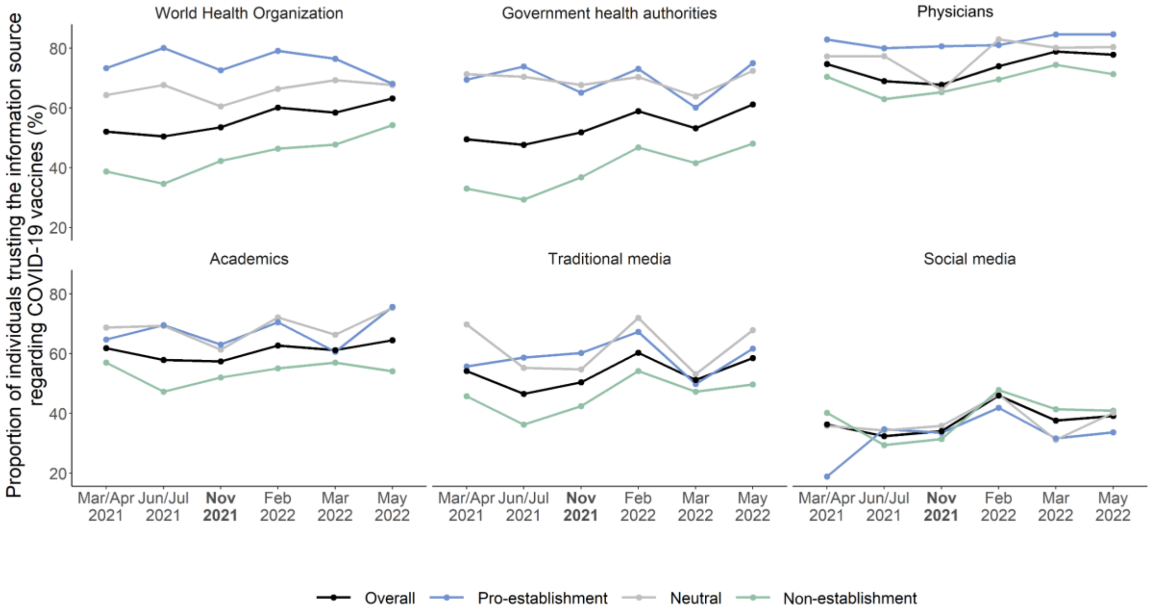

© 2023 Lun P et al. *JAMA Network Open.*

eFigure 8. Trends in COVID-19 vaccine confidence in Hong Kong, 2020–2022.

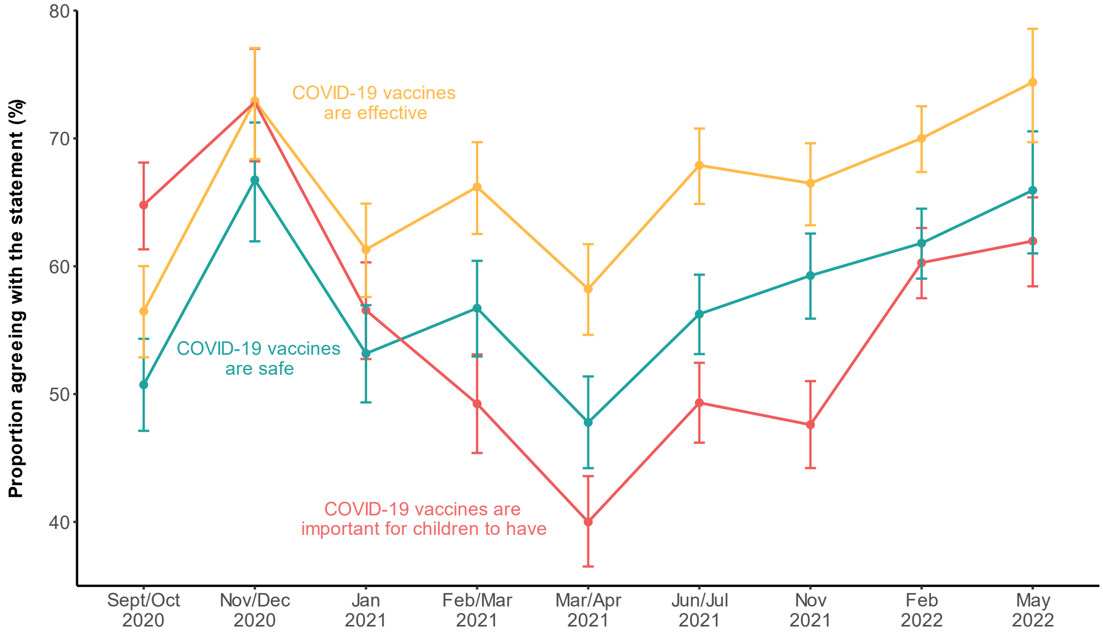

Error bars indicate the 95% confidence intervals.

© 2023 Lun P et al. *JAMA Network Open.*

eFigure 9. Trends in vaccine misconceptions in Hong Kong, 2021–2022.

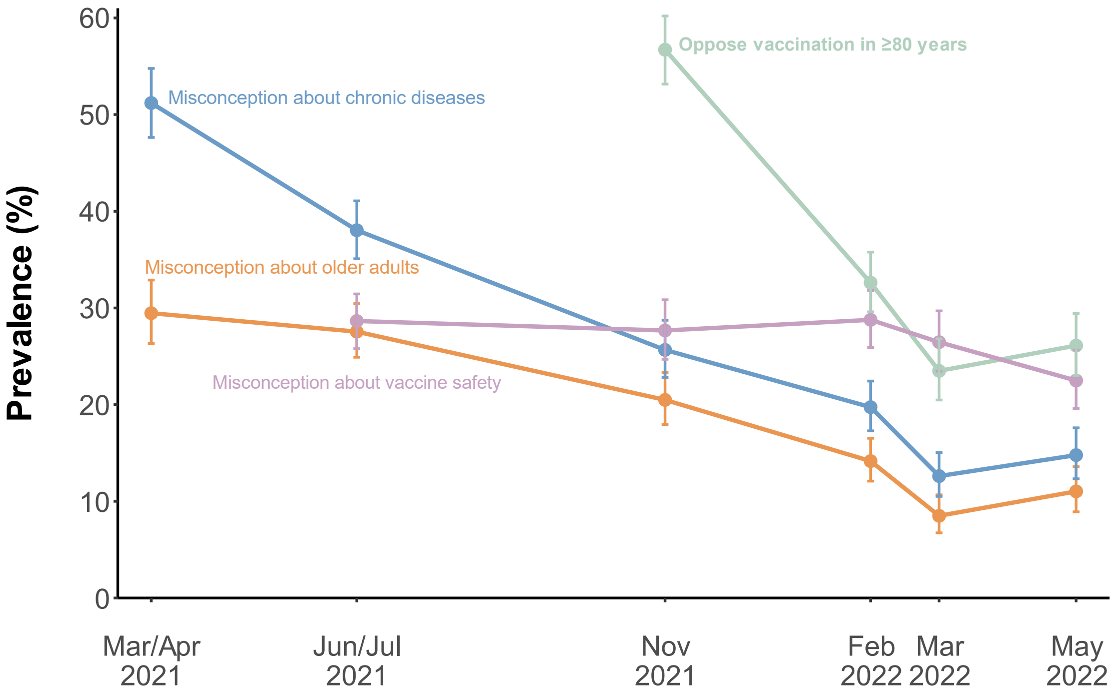

Trends in COVID-19 vaccine misconceptions in Hong Kong, 2021–2022. The three misconceptions about COVID-19 vaccines were assessed using the following statements: "Older individuals have a greater need for COVID-19 vaccination", "Individuals with more chronic diseases have a greater need for COVID-19 vaccination", and "COVID-19 vaccines are more harmful than COVID-19 infection. Disagreement with the first two statements and agreement with the last statement were considered as vaccine misconceptions. Error bars indicate the 95% confidence intervals.

© 2023 Lun P et al. *JAMA Network Open.*

eFigure 10. Population attributable fractions for factors, assessed in June–July 2021, associated with vaccine refusal and absolute reduction of vaccine refusal in November 2021.

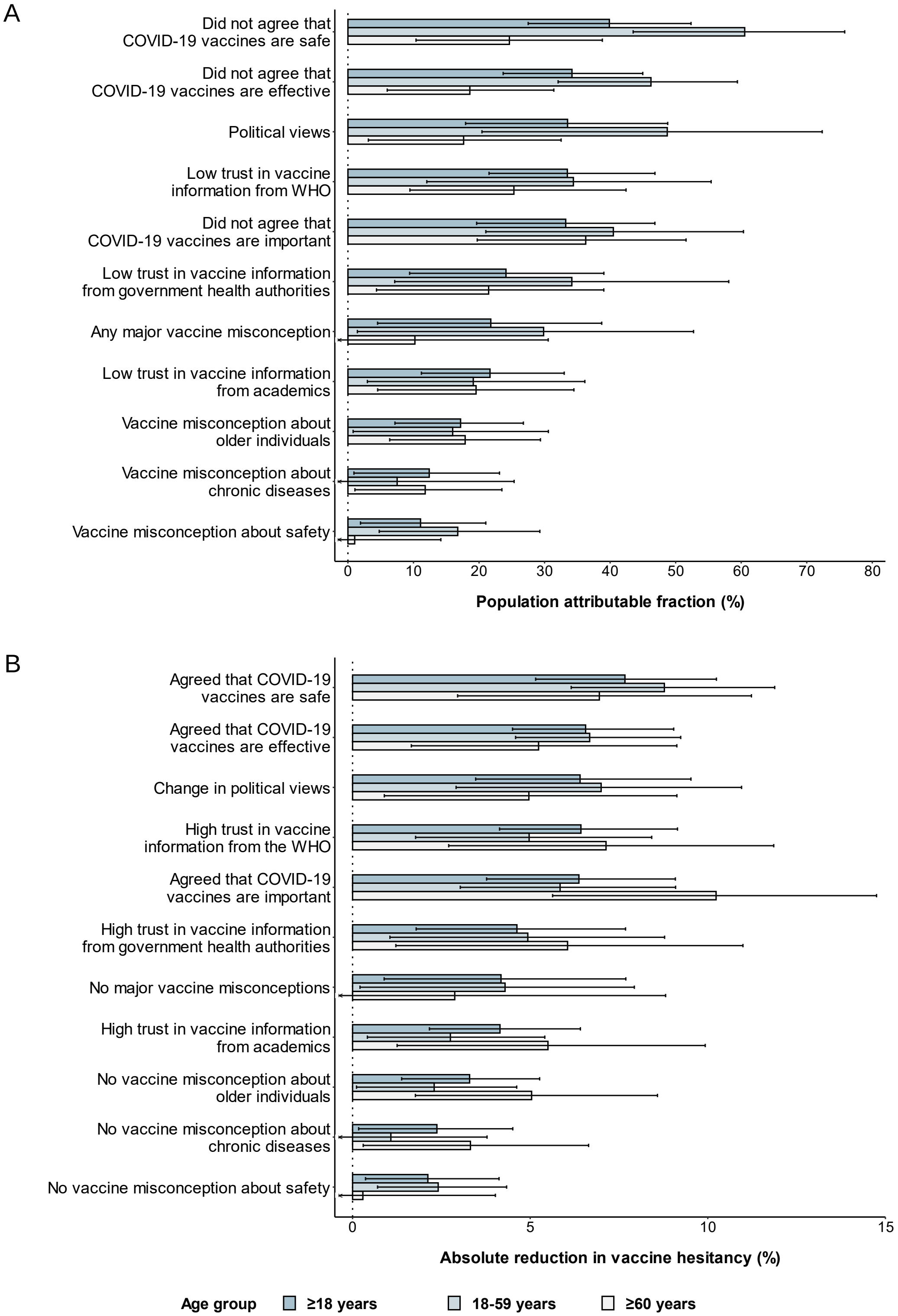

Error bars indicate the 95% confidence intervals.

© 2023 Lun P et al. *JAMA Network Open.*

eFigure 11. Population attributable fractions for factors, assessed in June–July 2021, associated with vaccine refusal and absolute reduction of vaccine refusal in November 2021.

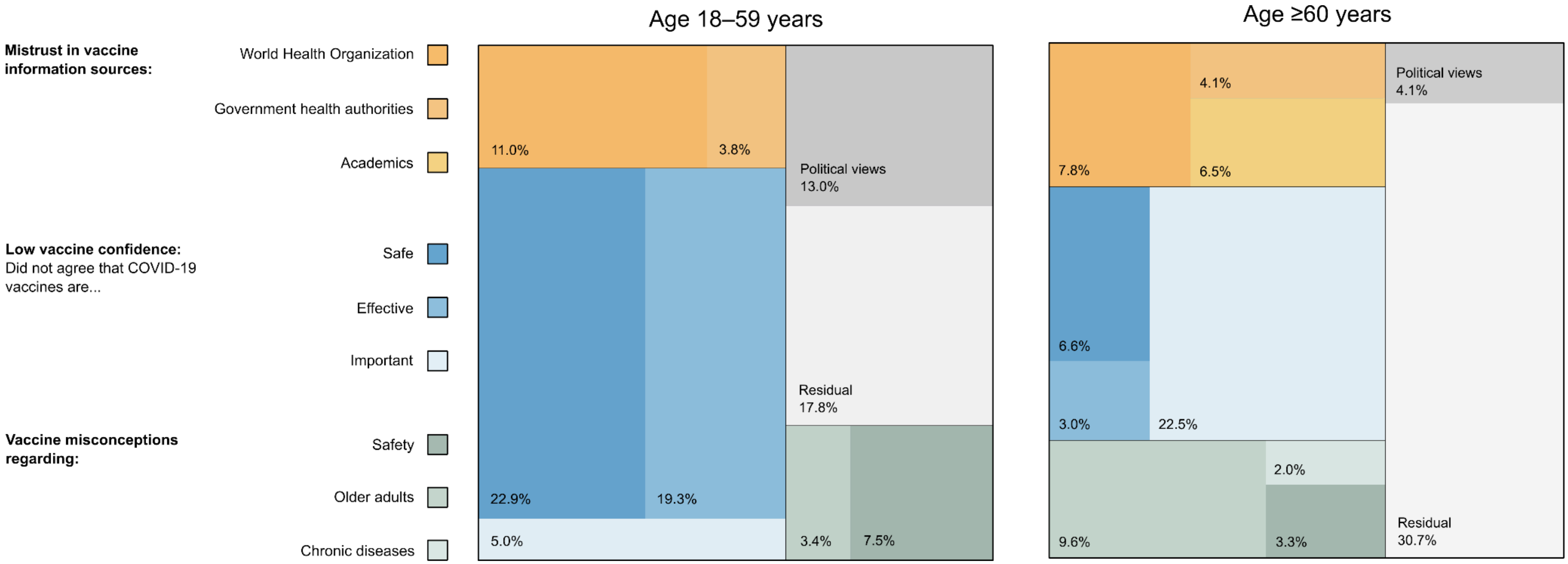

Sequential and average population attributable fraction of each determinant was calculated by modelling all determinants simultaneously. This may generate statistically non-significant negative associations between determinants and vaccine refusal, and consequently negative values of population attributable fraction. As such, values in the figure may not add up to 100%.

© 2023 Lun P et al. *JAMA Network Open.*

eTable 1. COVID-19 vaccine uptake among older adults before the first documented local Omicron transmission.

Regions	Fully vaccinated older adults[a] (aged ≥80)
New Zealand	97.1%
Japan	95.3%
Canada	95.0%
Australia	94.7%
England	93.3%
Singapore	91.0%
USA	84.2%[b]
South Korea	82.4%
Mainland China	40.0%
Hong Kong SAR	17.6%

Notes: Data were collected from Hong Kong Vaccination Dashboard, The State Council of the People's Republic of China, Australian Immunisation Register, Public Health Agency of Canada, Prime Minister's Office of Japan, Ministry of Health of New Zealand, Ministry of Health in Singapore, Central Disaster Management Headquarters of South Korean, National Health Service of England, and National Center for Immunization and Respiratory Diseases in the United States[26-34].

[a]Fully vaccinated refers to having completed the primary series of COVID-19 vaccines.

[b]Data included older adults aged ≥75 years.

© 2023 Lun P et al. *JAMA Network Open.*

eTable 2. List of outcome and exposures used in Hong Kong.

Variable category	Variable	Level/ Range	Wave/ Period
Willingness to vaccinate against COVID-19	Before launch of local vaccination program<br><br>Questions:<br>Version 1: If a vaccine to prevent coronavirus/COVID-19 was approved for use in Hong Kong and was available right now at no cost, would you agree to be vaccinated?<br><br>Version 2: Based on Hong Kong's current plan for COVID-19 vaccination programme, would you join the programme?	Ordinal: 0-No, 1-Yes<br><br>Recoding: Vaccination willingness: 1<br>Vaccine refusal: 0	11–14
Willingness to vaccinate against COVID-19	After launch of local vaccination program<br><br>Question:<br>Based on Hong Kong's current plan for COVID-19 vaccination programme, have you joined the programme?	Ordinal: 0-No and I don't plan to join the programme,<br>1-No but I plan to join the programme, 2-No but I have made an appointment for vaccination shots, 3-Yes, I have received the first dose of vaccine, 4-Yes, I have received two doses of vaccine,<br><br>Recoding: Vaccination willingness: 1–4; Vaccine refusal: 0	15–20
Willingness to vaccinate against COVID-19	Official daily number of adult COVID-19 vaccination appointments in Hong Kong	Range: 36–111,046	Feb 23rd, 2021 – May 30th, 2022
Acceptance of COVID-19 vaccines	The Hong Kong Government has established Advanced Purchase Agreement with the three following vaccines. We would like to learn about your thoughts on all these vaccines even though you might have already taken part in the COVID-19 vaccination programme:<br><br>Question:<br>The (first/second/third) vaccine is SinoVac vaccine (Coronavac). Would you be willing to receive this vaccine?	Binary: 0-No, 1-Yes	16
Acceptance of COVID-19 vaccines	Question:	Binary: 0-No, 1-Yes	16

© 2023 Lun P et al. *JAMA Network Open.*

	The (first/second/third) vaccine is the Fosun-BioNTech vaccine (Comirnaty). Would you be willing to receive this vaccine?		
Acceptance of COVID-19 vaccines	Question:<br>The (first/second/third) vaccine is the Oxford-AstraZeneca vaccine (Vaxzevria). Would you be willing to receive this vaccine?	Binary: 0-No, 1-Yes	16
Media reports on Adverse Events Following COVID-19 Immunisation (AEFIs)	Number of newspaper clippings from Hong Kong traditional newspaper publishers and online news media	Range: 0-77	Aug 1st, 2020 – May 30th, 2022
Political views and identities	2019/2020 social unrest<br><br>Question:<br>Do you think the extradition amendment bill should be passed?	Ordinal: 1- Neutral or No Comments, 2- Yes, 3-No<br><br>Recoding: Neutral: 1, Pro-establishment: 2, Non-establishment: 3	5–6
Political views and participation	2014 Occupy Central<br><br>Question:<br>In the past month, did you ever visit the protest sites?	Binary: 0-No, 1-Yes<br><br>Recoding: Pro-establishment: 0, Non-establishment: 1	3–4
Trust in COVID-19 vaccine information source	World Health Organization<br><br>Question:<br>On a scale of 1 to 10, how much do you trust World Health Organization (WHO) on COVID-19 vaccine related information?	Range: 1(having no trust at all)–10(having complete trust)<br><br>Recoding: Mistrust: ≤5, Trust: >6	15–20
Trust in COVID-19 vaccine information source	Government health authorities<br><br>Question:<br>On a scale of 1 to 10, how much do you trust Government departments and related institutions like FHB, HA, and CHP on COVID-19 vaccine related information?	Range: 1(having no trust at all)–10(having complete trust)<br><br>Recoding: Mistrust: ≤5, Trust: >6	15–20
Trust in COVID-19 vaccine information source	Physicians<br><br>Question:<br>On a scale of 1 to 10, how much do you trust your physician on COVID-19 vaccine related information?	Range: 1(having no trust at all)–10(having complete trust)<br><br>Recoding: Mistrust: ≤5, Trust: >6	15–20

© 2023 Lun P et al. *JAMA Network Open.*

Trust in COVID-19 vaccine information source	Academics<br><br>Question:<br>On a scale of 1 to 10, how much do you trust local public health or infectious disease academics on COVID-19 vaccine related information?	Range: 1(having no trust at all)–10(having complete trust)<br><br>Recoding: Mistrust: ≤5, Trust: >6	15–20
Trust in COVID-19 vaccine information source	Traditional media platforms<br><br>Question:<br>On a scale of 1 to 10, how much do you trust the traditional media platforms (e.g. television news, newspaper, radio stations) on COVID-19 vaccine related information?	Range: 1(having no trust at all)–10(having complete trust)<br><br>Recoding: Mistrust: ≤5, Trust: >6	15–20
Trust in COVID-19 vaccine information source	Social media platforms<br><br>Question:<br>On a scale of 1 to 10, how much do you trust the social media (e.g. Facebook, WhatsApp, Instagram, Telegram, WeChat, Twitter, Weibo, HKGolden, LIHKG, YouTube) on COVID-19 vaccine related information?	Range: 1(having no trust at all)–10(having complete trust)<br><br>Recoding: Mistrust: ≤5, Trust: >6	15–20
Trust in COVID-19 vaccine information source	Family/ friends<br><br>Question:<br>On a scale of 1 to 10, how much do you trust your family/friends on COVID-19 vaccine related information?	Range: 1(having no trust at all)–10(having complete trust)<br><br>Recoding: Mistrust: ≤5, Trust: >6	18–20
COVID-19 vaccine confidence	Effectiveness<br><br>Question:<br>To what extent do you agree with the following statement pertaining to COVID-19 vaccines?<br><br>"I think COVID-19 vaccines are effective."	Ordinal: Strongly agree; Somewhat agree; Neither agree nor disagree; Somewhat disagree; Strongly disagree	11–18,20
COVID-19 vaccine confidence	Safety<br><br>Question:<br>To what extent do you agree with the following statement pertaining to COVID-19 vaccines? "I think COVID-19 vaccines are safe."	Ordinal: Strongly agree, Somewhat agree, Neither agree nor disagree, Somewhat disagree, Strongly disagree	11–18,20
COVID-19 vaccine confidence	Importance of COVID-19 vaccines<br><br>Question:	Ordinal: Strongly agree, Somewhat agree, Neither agree nor disagree, Somewhat disagree, Strongly disagree	11–18,20

© 2023 Lun P et al. *JAMA Network Open.*

	To what extent do you agree with the following statement pertaining to COVID-19 vaccines? “I think COVID-19 vaccines are important for children to have.”		
COVID-19 vaccine misconceptions	Regarding older adults<br><br>Question:<br>To what extent do you agree with the following statement pertaining to COVID-19 vaccines? “Older individuals have a greater need for COVID-19 vaccination”	Ordinal: Strongly agree, Somewhat agree, Neither agree nor disagree, Somewhat disagree, Strongly disagree<br><br>Recoding: With misconception: Somewhat disagree, Strongly disagree; Without misconception: Strongly agree, Somewhat agree, Neither agree nor disagree	15–20
COVID-19 vaccine misconceptions	Regarding chronic diseases<br><br>Question:<br>To what extent do you agree with the following statement pertaining to COVID-19 vaccines?<br>“Individuals with more chronic diseases have a greater need for COVID-19 vaccination”	Ordinal: Strongly agree, Somewhat agree, Neither agree nor disagree, Somewhat disagree, Strongly disagree<br><br>Recoding: With misconception: Somewhat disagree, Strongly disagree; Without misconception: Strongly agree, Somewhat agree, Neither agree nor disagree	15–20
COVID-19 vaccine misconceptions	Regarding COVID-19 vaccine safety<br><br>Question:<br>To what extent do you agree with the following statement pertaining to COVID-19 vaccines?<br>“COVID-19 vaccines are more harmful than COVID-19 infection”	Ordinal: Strongly agree, Somewhat agree, Neither agree nor disagree, Somewhat disagree, Strongly disagree<br><br>Recoding:<br>With misconception: Strongly agree, Somewhat agree; Without misconception: Neither agree nor disagree, Somewhat disagree, Strongly disagree	15–20
COVID-19 vaccine misconceptions	Number of COVID-19 vaccine misconceptions	Range: 0–3	15–20
COVID-19 vaccine misconceptions	Oppose COVID-19 vaccination in ≥80 years<br><br>Question:<br>To your knowledge, should the people aged 80 or above receive COVID-19 vaccines available in Hong Kong?	Binary: Yes, No<br><br>Recoding: With misconception: No; With misconception: Yes	17–20
COVID-19 vaccine misconceptions	Information sources for misconceptions<br><br>Question:	7 levels: World Health Organization, Government health authorities, Physicians, Academics,	19

© 2023 Lun P et al. *JAMA Network Open.*

	What is the primary information source that informed this particular view?	Traditional media platforms, Social media platforms, Family/ friends	
Social factors	Decision making on COVID-19 vaccine uptake among older adults<br><br>Question:<br>Who were involved in your decision-making process of getting COVID-19 vaccines?	5 levels: Self, Family members, Physicians, Friends, Others	19
Socio-demographics	Sex	2 levels: Male, Female	-
Socio-demographics	Age	Ordinal: 20–39, 40–59, 60 and above	-
Socio-demographics	Education attainment	Ordinal: Primary, Secondary, Tertiary	-
Socio-demographics	Marital status	3 levels: Married, Never married, Widowed/ divorced/ separated	-
Socio-demographics	Employment status	3 levels: Employed, Unemployed, Economically inactive	-
Socio-demographics	Housing type	3 levels: Public rental housing, Subsidised home ownership scheme housing, Private permanent housing	-
Socio-demographics	Monthly household income (HKD)	Ordinal: <10,000; 10,000–19,999; 20,000–39,999; 40,000 and above	-

© 2023 Lun P et al. *JAMA Network Open.*

eTable 3. Demographic composition of wave 20 compared to 2016 Population By-census of Hong Kong.

Demographics	Hong Kong By-census	Wave 20 (Weighted)	Effect size
	No. (%)	No. (%)	Wave 20 vs. Census
Sex			
Female Sex	3076,902 (52.7)	571 (51.3)	0.03
Age group (years)			
20–39	1,884,500 (32.2)	335 (30.0)	0.05
40–59	2,303,268(39.4)	452 (40.6)	
60 and above	1,655,814 (28.3)	327 (29.4)	
Education attainment			
Primary	1,271,564 (21.8)	216 (19.4)	0.06
Secondary	2,593,749(44.4)	519 (46.6)	
Tertiary	1,978,269 (33.9)	378 (34.0)	
Marital status			
Married	3,605,859 (61.7)	708 (63.7)	0.05
Never married	1,522,327 (26.1)	268 (24.2)	
Widowed/ divorced/ separated	715,396 (12.2)	134 (12.1)	
Housing type			
Public rental housing	1,781,209 (30.2)	343 (31.1)	0.05
Subsidised home ownership scheme housing	1,003,096 (17.0)	204 (18.5)	
Private permanent housing	3,111,832 (52.8)	556 (50.4)	
Monthly household income (HKD)			
<10,000	490,496 (19.6)	202 (18.6)	0.06
10,000–19,999	544,291 (21.7)	216 (19.9)	
20,000–39,999	700,557 (27.9)	318 (29.3)	
40,000 and above	772,678 (30.8)	349 (32.2)	
Cohen's w effect size = small 0.1; medium 0.3; large 0.5.			

© 2023 Lun P et al. *JAMA Network Open.*

eTable 4. Interrupted time-series analyses of intervention measures and daily COVID-19 vaccination appointments.

	Age ≥18 years	Age 18–59 years	Age ≥60 years
MA (Lag 1)	**-20.7% (-28.4, -12.1)**	**-16.7% (-25.1, -7.3)**	**-21.7% (-29.2, -13.4)**
MA (Lag 2)	**-12.0% (-20.8, -2.2)**	**-15.2% (-24.1, -5.1)**	**-12.2% (-19.9, -3.6)**
MA, Seasonal (Lag 1)	**-61.4% (-63.3, -59.3)**	**-63.2% (-65.3, -61.0)**	**-57.4% (-60.6, -53.9)**
Lottery-based incentives	22.2% (-12.5, 70.6)	21.5% (-12.2, 68.0)	27.3% (-15.4, 91.5)
Workplace mandates	**62.2% (9.9, 139.2)**	**73.7% (20.2, 150.9)**	24.4% (-23.1, 101.2)
Vaccine pass	**124.8% (65.9, 204.6)**	**130.9% (72.2, 209.7)**	**93.5% (32.5, 182.4)**
Lunar New Year	**-54.9% (-67.3, -37.7)**	**-50.8% (-64.0, -32.8)**	**-61.3% (-74.0, -42.4)**
Omicron surge	**93.3% (42.2, 162.8)**	**43.8% (6.1, 94.8)**	**106.8% (42.2, 200.6)**
Reopening of premises under the vaccine pass	**81.0% (34.0, 144.4)**	15.4% (-13.8, 54.5)	**111.6% (47.5, 203.6)**
Abbreviations: MA = moving average. Coefficient refers to the percent change in vaccine appointment numbers associated with corresponding intervention measures; 95% confident intervals of the percent change are presented in brackets. Bolded are statistically significant (p<0.05).			

© 2023 Lun P et al. *JAMA Network Open.*

eTable 5. Associations of political views, trust in information sources, vaccine misconceptions, and vaccine confidence in June–July, 2021 with vaccine refusal in the general population (≥18 years) and older adults (≥60 years) in November 2021.

**Variables**	**≥18 years**	**≥60 years**
	**aIRR (95% CI)**	**aIRR (95% CI)**
Political views during 2019 Social Unrest [a]		
Pro-establishment	1(ref)	1(ref)
Neutral	1.89 (0.91, 3.92)	1.61 (0.85, 3.03)
Non-establishment	**3.26 (1.63, 6.51)**	**2.10 (1.15, 3.83)**
Trust in COVID-19 vaccine information sources[b]		
World Health Organization		
Trust	1(ref)	1(ref)
Mistrust	**1.90 (1.41, 2.55)**	**1.69 (1.14, 2.52)**
Government health authorities		
Trust	1(ref)	1(ref)
Mistrust	**1.56 (1.14, 2.14)**	**1.60 (1.09, 2.35)**
Academics		
Trust	1(ref)	1(ref)
Mistrust	**1.66 (1.27, 2.18)**	**1.59 (1.09, 2.30)**
Physicians		
Trust	1(ref)	1(ref)
Mistrust	1.29 (0.98, 1.69)	1.38 (0.95, 2.01)
Traditional media		
Trust	1(ref)	1(ref)
Mistrust	1.34 (1.00, 1.80)	1.15 (0.80, 1.67)
Social media		
Trust	1(ref)	1(ref)
Mistrust	1.17 (0.86, 1.59)	1.39 (0.93, 2.08)
COVID-19 vaccine misconceptions[b]		
Misconception about older adults		
No	1(ref)	1(ref)
Yes	**1.67 (1.27, 2.20)**	**2.03 (1.34, 3.06)**
Misconception about chronic diseases		
No	1(ref)	1(ref)
Yes	**1.37 (1.03, 1.83)**	1.46 (0.98, 2.16)
Misconception about safety		
No	1(ref)	1(ref)
Yes	**1.37 (1.05, 1.79)**	1.08 (0.73, 1.61)
Number of misconceptions		
None	1 (ref)	1 (ref)
1	1.10 (0.78, 1.55)	0.91 (0.57, 1.43)
2	**1.71 (1.18, 2.48)**	1.57 (0.96, 2.58)
3	**2.08 (1.35, 3.19)**	**2.65 (1.54, 4.55)**
COVID-19 vaccine confidence[b]		
COVID-19 vaccines are effective		
Agree	1 (ref)	1 (ref)
Did not agree	**2.48 (1.86, 3.29)**	**1.66(1.12, 2.47)**
COVID-19 vaccines are safe		
Agree	1 (ref)	1 (ref)
Did not agree	**2.35 (1.74, 3.16)**	**1.80(1.18, 2.75)**
COVID-19 vaccines are important for children to have		
Agree	1 (ref)	1 (ref)
Did not agree	**1.86 (1.36, 2.55)**	**2.23(1.47, 3.40)**

Abbreviations: aIRR=adjusted incidence rate ratio; ref= reference level; 95% CI=95% confidence interval.
Notes: [a]Robust Poisson regression models adjusted for sociodemographics; [b]Robust Poisson regression models adjusted for sociodemographics and political views. Bolded are statistically significant (p<0.05).

© 2023 Lun P et al. *JAMA Network Open.*

eTable 6. Associations of political views during 2014 Occupy Central with vaccine refusal over COVID-19 pandemic.

	**Prevalence of vaccine refusal**	
	**(95% CI)**	**aIRR**
Political participation during the 2014 Occupy Central		
No	31.9% (29.2, 38.4)	1 (ref)
Yes	41.1% (38.4, 43.8)	1.29 (1.16, 1.43)

Abbreviations: aIRR=adjusted incidence rate ratio; ref= reference level; 95% CI=95% confidence interval. Notes: Generalised estimating equations adjusted for sociodemographics and with an independent correlation matrix. Bolded are statistically significant ($p<0.05$).

© 2023 Lun P et al. *JAMA Network Open.*

eTable 7. Association of political views during 2019 Social Unrest with trust in COVID-19 vaccine information sources, vaccine misconceptions, and vaccine confidence in June 2021.

	**Non-establishment vs pro-establishment**	**Neutral vs non-establishment**
	aIRR (95% CI)	aIRR (95% CI)
Trust in COVID-19 vaccine information sources		
World Health Organization	**2.67 (1.87, 3.81)**	1.40 (0.91, 2.17)
Government health authorities	**2.29 (1.73, 3.03)**	1.06 (0.75, 1.51)
Academics	**1.55 (1.15, 2.09)**	0.94 (0.65, 1.36)
Physicians	**1.66 (1.09, 2.54)**	1.04 (0.63, 1.71)
Traditional media	**1.56 (1.20, 2.02)**	1.19 (0.88, 1.62)
Social media	1.06 (0.89, 1.25)	1.04 (0.83, 1.29)
COVID-19 vaccine misconceptions		
Misconception about older adults	**2.68 (1.61, 4.45)**	1.63 (0.92, 2.88)
Misconception about chronic diseases	**1.84 (1.29, 2.62)**	1.34 (0.88, 2.03)
Misconception about vaccine safety	**2.02 (1.34, 3.05)**	1.39 (0.84, 2.27)
COVID-19 vaccine confidence		
Did not agree that COVID-19 vaccines are effective	**4.38 (2.17, 8.87)**	**2.97 (1.43, 6.17)**
Did not agree that COVID-19 vaccines are safe	**4.05 (2.40, 6.81)**	**2.47 (1.42, 4.30)**
Did not agree that COVID-19 vaccines are important for children	**2.74 (1.86, 4.05)**	**2.01 (1.34, 3.02)**

Abbreviations: aIRR = adjusted incidence rate ratio; 95% CI = 95% confidence interval. Robust Poisson regression models adjusted for sociodemographics. Bolded are statistically significant ($p<0.05$).

eTable 8. Association between trust in COVID-19 vaccine information sources in June–July, 2021 and endorsement of vaccine misconceptions and COVID-19 vaccine confidence in November 2021.

	**Misconception about older adults**	**Misconception about chronic diseases**	**Misconception about vaccine safety**	**COVID-19 vaccines are effective**	**COVID-19 vaccines are safe**	**COVID-19 vaccines are important for children to have**
	aIRR (95% CI)	aIRR (95% CI)	aIRR (95% CI)	aIRR (95% CI)	aIRR (95% CI)	aIRR (95% CI)
World Health Organization						
Trust	1 (Ref)	1 (Ref)	1 (Ref)	1 (Ref)	1 (Ref)	1 (Ref)
Mistrust	**1.64 (1.21, 2.21)**	1.29 (0.99, 1.68)	1.25 (0.96, 1.61)	**1.61 (1.30, 2.00)**	**1.69 (1.40, 2.05)**	**1.53 (1.31, 1.79)**
Government health authorities						
Trust	1 (Ref)	1 (Ref)	1 (Ref)	1 (Ref)	1 (Ref)	1 (Ref)
Mistrust	**1.95 (1.40, 2.73)**	**1.43 (1.07, 1.90)**	1.05 (0.81, 1.36)	**2.03 (1.59, 2.59)**	**1.86 (1.52, 2.28)**	**1.48 (1.24, 1.77)**
Academics						
Trust	1 (Ref)	1 (Ref)	1 (Ref)	1 (Ref)	1 (Ref)	1 (Ref)
Mistrust	**1.61 (1.22, 2.14)**	1.13 (0.88, 1.46)	1.12 (0.88, 1.43)	**1.56 (1.27, 1.91)**	**1.54 (1.31, 1.81)**	**1.51 (1.31, 1.73)**
Physicians						
Trust	1 (Ref)	1 (Ref)	1 (Ref)	1 (Ref)	1 (Ref)	1 (Ref)
Mistrust	1.13 (0.85, 1.50)	0.98 (0.77, 1.25)	1.10 (0.86, 1.41)	**1.53 (1.26, 1.86)**	**1.31 (1.11, 1.53)**	**1.20 (1.05, 1.37)**
Traditional media						
Trust	1 (Ref)	1 (Ref)	1 (Ref)	1 (Ref)	1 (Ref)	1 (Ref)
Mistrust	**1.64 (1.23, 2.19)**	1.14 (0.90, 1.45)	1.06 (0.84, 1.34)	**1.79 (1.43, 2.23)**	**1.50 (1.25, 1.80)**	**1.25 (1.09, 1.44)**
Social media						
Trust	1 (Ref)	1 (Ref)	1 (Ref)	1 (Ref)	1 (Ref)	1 (Ref)
Mistrust	0.97 (0.73, 1.28)	0.86 (0.67, 1.10)	0.82 (0.64, 1.05)	**1.48 (1.16, 1.89)**	**1.39 (1.14, 1.70)**	**1.21 (1.04, 1.41)**

Abbreviations: aIRR = adjusted incidence rate ratio; Ref = reference level; 95% CI = 95% confidence interval.
Notes: Robust Poisson regression models adjusted for sociodemographics and political views. Bolded are statistically significant ($p<0.05$).

© 2023 Lun P et al. *JAMA Network Open.*

eTable 9. Direct and indirect effects of political views on vaccine refusal in November, 2021 via mistrust in vaccine information sources, vaccine misconceptions and vaccine confidence in June–July, 2021.

	**Exposure-mediator***	**Mediator-outcome**	**NDE**	**TNIE**	**TE**	**Proportion mediated**
	aIRR (95% CI)	aIRR (95% CI)	aIRR (95% CI)	aIRR (95% CI)	aIRR (95% CI)	Proportion (95% CI)
			1.39 (0.95, 1.93)	**1.73 (1.59, 2.68)**	**2.41 (1.92, 4.01)**	72.5% (61.7, 100.0)
Mistrust in COVID-19 vaccine information sources	-	-	-	-	-	-
World Health Organization	**1.57 (1.20, 2.06)**	1.36 (1.02, 1.81)	-	-	-	-
Government health authorities	**1.70 (1.25, 2.32)**	1.30 (0.93, 1.83)	-	-	-	-
Academics	**1.53 (1.19, 1.95)**	0.97 (0.70, 1.35)	-	-	-	-
COVID-19 vaccine misconceptions	-	-	-	-	-	-
Misconception about older adults	**2.10 (1.69, 2.61)**	1.34 (0.94, 1.90)	-	-	-	-
Misconception about chronic diseases	**2.21 (1.78, 2.73)**	0.91 (0.63, 1.33)	-	-	-	-
Misconception about safety	**1.61 (1.29, 2.00)**	1.21 (0.85, 1.73)	-	-	-	-
COVID-19 vaccine confidence	-	-	-	-	-	-
Did not agree that vaccines are effective	**2.04 (1.64, 2.54)**	**1.54 (1.06, 2.24)**	-	-	-	-
Did not agree that vaccines are safe	**1.83 (1.40, 2.39)**	**1.59 (1.13, 2.23)**	-	-	-	-
Did not agree that vaccines are important for children	1.66 (1.36, 2.03)	1.22 (0.87, 1.72)	-	-	-	-

Abbreviations: aIRR=adjusted incidence rate ratio; 95% CI=95% confidence interval; NDE= Natural direct effects; TNIE=total natural indirect effects; TE=total effects. Models adjusted for age, sex, education attainment, marital status, employment status, and monthly household income.

© 2023 Lun P et al. *JAMA Network Open.*

eTable 10. Social influences for decision-making on COVID-19 vaccine uptake among older adults, February 2022.

**Social influences**	**Proportion (%)**
Family members	54.7%
Physicians	30.2%
Friends	22.0%
Others	4.4%
Notes: Respondents could choose more than one option.	

© 2023 Lun P et al. *JAMA Network Open.*

eTable 11. Information source for misconceptions regarding priority groups for COVID-19 vaccination, March 2022.

Information source for COVID-19 vaccine misconception	Proportion (95% CI)
Misconception regarding older adults	
Social media	28.3 (18.7, 40.6)
Physicians	20.5 (12.4, 31.9)
Government health authorities	14.2 (7.7, 24.6)
Family/friends	10.6 (5.2, 20.5)
Others	9.4 (4.0, 20.4)
Traditional media	7.0 (2.5,18)
World Health Organization	6.6 (2.7, 15.3)
Academics	3.4 (0.7, 14.1)
Misconception regarding chronic diseases	
Social media	22.8 (15.7, 32.0)
Family/friends	21.4 (14.2, 30.8)
Physicians	18.6 (12.4, 27.1)
World Health Organization	10.0 (5.7, 16.8)
Traditional media	9.0 (4.3, 17.8)
Government health authorities	7.2 (3.6, 14.1)
Academics	6.5 (2.9, 13.8)
Others	4.5 (2.2, 9.1)
Oppose COVID-19 vaccination in ≥80 years	
Family/friends	23.4 (17.4, 30.7)
Social media	18.7 (13.4, 25.4)
Physicians	17.2 (12.0, 24.1)
Traditional media	9.6 (5.9, 15.5)
Others	9.6 (5.9, 15.2)
Government health authorities	7.7 (4.5, 13.1)
Academics	7.3 (4.3, 12.2)
World Health Organization	6.4 (3.6, 11.1)
Abbreviations: 95% CI=95% confidence interval.	

© 2023 Lun P et al. *JAMA Network Open.*

eReferences

1. Subramaniam M, Abdin E, Vaingankar JA, et al. Tracking the mental health of a nation: prevalence and correlates of mental disorders in the second Singapore mental health study. *Epidemiol Psychiatr Sci*. 2019;29(e29):1–10. doi:10.1017/S2045796019000179
2. The American Association for Public Opinion Research. *Standard Definitions Report*. 2023. Accessed August 1 2023. https://aapor.org/standards-and-ethics/standard-definitions/
3. Larson HJ, de Figueiredo A, Xiahong Z, et al. The state of vaccine confidence 2016: global insights through a 67-country survey. *EBioMedicine*. 2016;12:295–301. doi:10.1016/j.ebiom.2016.08.042
4. de Figueiredo A, Simas C, Karafillakis E, Paterson P, Larson HJ. Mapping global trends in vaccine confidence and investigating barriers to vaccine uptake: a large-scale retrospective temporal modelling study. *Lancet*. 2020;396(10255):898–908. doi:10.1016/S0140-6736(20)31558-0
5. The Government of the Hong Kong Special Administrative Region. COVID-19 vaccination scheme starts. Updated Feb 26. Accessed April 8, 2022. https://www.news.gov.hk/eng/2021/02/20210226/20210226_174848_473.html?type=category&name=covid19&tl=t
6. Hong Kong International Airport. Airport Authority launches air ticket lucky draw to promote vaccination. Hong Kong International Airport. Updated May 26. Accessed Apr 10, 2022. https://www.hongkongairport.com/en/media-centre/press-release/2021/pr_1547
7. Leung K. Coronavirus: lottery to win HK$10.8 million flat opens on June 15, businesses offer range of cash perks for vaccination. South China Morning Post. Updated Jun 2. Accessed Apr 8, 2022. https://www.scmp.com/news/hong-kong/society/article/3135806/coronavirus-lottery-win-hk108-million-flat-opens-june-15
8. The Government of the Hong Kong Special Administrative Region. Vaccine bubble to be expanded. Updated Dec 31. Accessed April 8, 2022. https://www.news.gov.hk/eng/2021/12/20211231/20211231_170939_623.html?type=category&name=health&tl=t
9. The Government of the Hong Kong Special Administrative Region. Social distancing rules to be relaxed. Accessed June 8, 2022. https://www.news.gov.hk/eng/2022/04/20220414/20220414_121238_430.html?type=category&name=health&tl=t
10. Wisers Information Limited. WiseNews
11. Drug office of Department of Health. *Guidance for Healthcare Professionals - Reporting of Adverse Event Following Immunization of COVID-19 Vaccine* 2022. https://www.drugoffice.gov.hk/eps/do/en/doc/Guidance_for_HCP_(COVID_finalize).pdf
12. Cohen J. *Statistical power analysis for the behavioral sciences*. 2nd ed. L. Erlbaum Associates; 1988.
13. Schaffer AL, Dobbins TA, Pearson SA. Interrupted time series analysis using autoregressive integrated moving average (ARIMA) models: a guide for evaluating large-scale health interventions. *BMC Med Res Methodol*. 2021;21(1):58. doi:10.1186/s12874-021-01235-8
14. Shi B, Choirat C, Coull BA, VanderWeele TJ, Valeri L. CMAverse: A suite of functions for reproducible causal mediation analyses. *Epidemiology*. 2021;32(5):e20–e22. doi:10.1097/EDE.0000000000001378

© 2023 Lun P et al. *JAMA Network Open.*

15. VanderWeele TJ, Tchetgen Tchetgen EJ. Mediation analysis with time varying exposures and mediators. *J R Stat Soc Series B Stat Methodol*. 2017;79(3):917–938. doi:10.1111/rssb.12194
16. Heymans MW, Eekhout I. Rubin's rules. *Applied missing data with SPSS and (R)Studio*. 2019.
17. McMenamin ME, Nealon J, Lin Y, et al. Vaccine effectiveness of one, two, and three doses of BNT162b2 and CoronaVac against COVID-19 in Hong Kong: a population-based observational study. *Lancet Infect Dis*. 2022;22(10):1435-1443. doi:10.1016/S1473-3099(22)00345-0
18. Collie S, Champion J, Moultrie H, Bekker LG, Gray G. Effectiveness of BNT162b2 vaccine against Omicron variant in South Africa. *N Engl J Med*. 2022;386(5):494–496. doi:10.1056/NEJMc2119270
19. Wang K, Jia Z, Bao L, et al. Memory B cell repertoire from triple vaccinees against diverse SARS-CoV-2 variants. *Nature*. 2022;603(7903):919–925. doi:10.1038/s41586-022-04466-x
20. Di Maso M, Bravi F, Polesel J, et al. Attributable fraction for multiple risk factors: Methods, interpretations, and examples. *Stat Methods Med Res*. 2020;29(3):854–865. doi:10.1177/0962280219848471
21. Ferguson J. *Averisk: calculation of average population attributable fractions and confidence intervals*. R Foundation for Statistical Computing; 2017.
22. Brady AR. Adjusted population attributable fractions from logistic regression. *Stata Technical Bulletin*. 1998;7(42):1–47.
23. White IR, Royston P, Wood AM. Multiple imputation using chained equations: issues and guidance for practice. *Stat Med*. 2011;30(4):377–399. doi:https://doi.org/10.1002/sim.4067
24. Schomaker M, Heumann C. Bootstrap inference when using multiple imputation. *Stat Med*. 2018;37(14):2252–2266. doi:10.1002/sim.7654
25. Kolenikov S. Resampling variance estimation for complex survey data. *Stata J*. 2010;10(2):165–199.
26. The Government of the Hong Kong Special Administrative Region. Hong Kong Vaccination Dashboard. Accessed March 31, 2022. https://www.covidvaccine.gov.hk/en/dashboard
27. Department of Health of the Australian Government. COVID-19 vaccination – vaccination data – 10 March 2022. Updated Mar 10. Accessed Apr 7,, 2022. https://www.health.gov.au/resources/publications/covid-19-vaccination-vaccination-data-10-march-2022
28. Government of Canada. COVID-19 vaccination in Canada. Government of Canada. Updated Feb 27. Accessed Apr 8, 2022. https://health-infobase.canada.ca/covid-19/vaccination-coverage/
29. Reuters. China vaccinates over 80% of its people against Covid-19. Dec 11. https://timesofindia.indiatimes.com/world/china/china-vaccinates-over-80-of-its-people-against-covid-19/articleshow/88224900.cms
30. Prime Minister's Office of Japan. 新型コロナワクチンについて. Prime Minister's Office of Japan,. Accessed Dec 31,, 2021. https://www.kantei.go.jp/jp/headline/kansensho/vaccine.html
31. Ministry of Health of New Zealand. COVID-19: Vaccine data. Ministry of Health of New Zealand,. Accessed Dec 31,, 2021. https://www.health.govt.nz/covid-19-novel-coronavirus/covid-19-data-and-statistics/covid-19-vaccine-data

© 2023 Lun P et al. *JAMA Network Open.*

32. Ministry of Health of Singapore. Vaccination statistics. Ministry of Health of Singapore. Updated Mar 7. Accessed Apr 8, 2022. https://www.moh.gov.sg/covid-19/vaccination/statistics
33. National Health Service England. COVID-19 vaccinations. National Health Service England. Accessed Apr 6, 2022. https://www.england.nhs.uk/statistics/statistical-work-areas/covid-19-vaccinations/
34. Centers for Disease Control and Prevention. COVID-19 vaccination and case trends by age group, United States. Updated Mar 7. Accessed Dec 31, 2021. https://data.cdc.gov/Vaccinations/COVID-19-Vaccination-and-Case-Trends-by-Age-Group-/gxj9-t96f

© 2023 Lun P et al. *JAMA Network Open.*
