# Supplementary material for: COVID-19 Vaccination Willingness and Reasons for Vaccine Refusal
Source: JAMA Netw Open. 2023 Oct 19;6(10):e2337909. doi: 10.1001/jamanetworkopen.2023.37909 (PMC10587797; doi:10.1001/jamanetworkopen.2023.37909)
Supplement: Supplement 2. — Data Sharing Statement [file jamanetwopen-e2337909-s002.pdf]

Data Sharing Statement

Lun. COVID-19 Vaccination Willingness and Reasons for Vaccine Refusal in Hong Kong. *JAMA Netw Open*. Published October 19, 2023. doi:10.1001/jamanetworkopen.2023.37909

Data
Data available: Yes
Data types: Deidentified participant data, Data dictionary
How to access data: Data used in this analysis can be obtained upon request made to the corresponding author (nimy@hku.hk).
When available: With publication

Supporting Documents
Document types: Statistical/analytic code
How to access documents: Codes used in this analysis can be obtained upon request made to the corresponding author (nimy@hku.hk).
When available: With publication

Additional Information
Who can access the data: Researchers whose proposed use of the data has been approved
Types of analyses: for any purpose
Mechanisms of data availability: After approval of a proposal, with a signed data access agreement
